# Mortality in older adults with frequent alcohol consumption and use of drugs with addiction potential – The Nord Trøndelag Health Study 2006-2008 (HUNT3), Norway, a population-based study

**DOI:** 10.1371/journal.pone.0214813

**Published:** 2019-04-16

**Authors:** Kjerstin Tevik, Geir Selbæk, Knut Engedal, Arnfinn Seim, Steinar Krokstad, Anne-S Helvik

**Affiliations:** 1 Norwegian National Advisory Unit on Ageing and Health, Vestfold Hospital Trust, Tønsberg, Norway; 2 General Practice Research Unit, Department of Public Health and Nursing, Faculty of Medicine and Health Sciences, Norwegian University of Science and Technology (NTNU), Trondheim, Norway; 3 The Research Centre for Age-related Functional Decline and Disease, Innlandet Hospital Trust, Ottestad, Norway; 4 Faculty of Medicine, Institute of Health and Society, University of Oslo, Oslo, Norway; 5 Department of Geriatric Medicine, Oslo University Hospital, Oslo, Norway; 6 HUNT Research Centre, Department of Public Health and Nursing, Faculty of Medicine and Health Sciences, Norwegian University of Science and Technology, (NTNU), Levanger, Norway; 7 Psychiatric Department, Levanger Hospital, Nord-Trøndelag Hospital Trust, Levanger, Norway; 8 St. Olavs University Hospital, Sluppen, Trondheim, Norway; University of British Columbia, CANADA

## Abstract

**Background:**

The aim of this study was to investigate whether frequent drinking, use of drugs with addiction potential and the possible combination of frequent drinking and use of prescribed drugs with addiction potential were associated with all-cause mortality in older adults.

**Methods:**

We used data from the Nord-Trøndelag Health Study (HUNT3 2006–08), a population-based study in Norway. A total of 11,545 (6,084 women) individuals 65 years and older at baseline participated. We assessed frequent drinking (≥ 4 days a week), occasional drinking (i.e. a few times a year), never drinking and non-drinking in the last year. Drugs with addiction potential were defined as at least one prescription of benzodiazepines, z-hypnotics or opioids during one year for a minimum of two consecutive years between 2005 and 2009. This information was drawn from the Norwegian Prescription Database. The main outcome was all-cause mortality with information drawn from the Norwegian Cause of Death Registry. Follow-up continued until death or latest at 31 December 2013. Logistic regression analyses were used to investigate all-cause mortality since date of study entry and exact age at time of death was unknown.

**Results:**

The adjusted logistic regression analyses showed that frequent drinking was not associated with all-cause mortality compared to occasional drinking. Men who reported to be never drinkers and non-drinkers in the last year had higher odds of mortality compared to those who drank occasionally. Use of prescribed drugs with addiction potential was associated with increased mortality in men, but not in women. No association was found between the possible combination of frequent drinking and use of prescribed drugs with addiction potential and mortality.

**Conclusion:**

Neither frequent drinking nor the possible combination of frequent drinking and use of prescribed drugs with addiction potential were associated with all-cause mortality in older women and men. Use of prescribed drugs with addiction potential was associated with higher odds of mortality in men. This finding should lead to more caution in prescribing drugs with addiction potential to this group.

## Introduction

Alcohol consumption has increased in recent decades among older adults in Western countries [1–3], and it is important to clarify the influence alcohol might have on the health of older adults [4]. Older adults are more sensitive to alcohol due to age-related physiological changes and may be more negatively affected by alcohol use compared to younger adults [4, 5]. Furthermore, older adults are more likely than younger adults to have chronic disorders and take multiple medications that can interact negatively with alcohol [4].

As in the general population [6–9], a J-shape association between alcohol consumption and mortality has been observed among middle-aged and older adults [10–16] showing the lowest mortality in individuals with low-to-moderate alcohol consumption and highest among abstainers and individuals with heavy alcohol consumption. The lower mortality risk found in low-to-moderate alcohol consumption may be due to the reference category used, i.e. use of abstainers [14] or non-drinkers [10, 15] who may introduce bias due to their poorer health [17, 18]. Abstainers and non-drinkers may include former drinkers who have quit drinking because of health problems [17, 19]. Thus, the survival effect for low-to-moderate consumption may be a result of confounding, also called abstainer bias [19]. It might be more reasonable to use infrequent or occasional alcohol consumption as reference category [20]. A meta-analysis of mortality independent of age found no reduction in mortality for low-volume alcohol consumption when using occasional alcohol consumption as a reference category [18]. A similar finding was confirmed in a recent meta-analysis [21].

Several studies have shown an association between heavy alcohol consumption and increased all-cause mortality in middle-aged and older adults, both when compared to abstaining from consumption [14, 16] and low-to-moderate alcohol consumption [13, 22–26]. However, this finding is inconsistent [27–30]. Heavy alcohol consumption has also been found to be related to increased mortality from cardiovascular disease [23] and alcohol-related cancer [21, 22]. Furthermore, heavy alcohol consumption is associated with a number of medical conditions, such as heart failure [31], hypertension [31], cardiac arrhythmia [21], stroke [21], liver disease [21], cancer [21], dementia [32] and mental health problems (depression, suicide, cognitive impairment) [33–35] all of which might increase mortality risk. In general, comparison of mortality in relation to alcohol consumption in studies of middle- aged and older adults are complicated due to the use of different alcohol measures, definitions and reference categories [10–16, 22–30].

Drugs with addiction potential (benzodiazepines, z-hypnotics and opioids) are used widely among older adults in Western countries [36–38] and often for conditions such as insomnia [39], anxiety [39] and chronic pain [40]. As a person ages, the pharmacokinetics and pharmacodynamics of drugs are altered, which result in prolonged and increased effects of these drugs [39]. The higher prevalence of comorbidity and polypharmacy in older adults increases the risk of adverse drug interactions [40].

Some studies have shown that use of BZD (anxiolytics, sedatives and hypnotics) and z-hypnotics in older adults is associated with increased mortality [39, 41, 42], while others have not found this association [43–47]. Higher risk of mortality might be related to altered consciousness [46], respiratory depression [48] and pneumonia [49]. Furthermore, BZD and z-hypnotics are associated with dementia [50], Alzheimer`s disease [51], depression [52], falls and hip fractures [53, 54], accidents and car crashes [53, 55], all of which increase the risk of mortality. It remains unclear whether some of these associations are causal or confounded by reverse causality [41, 42, 56, 57] as BZD frequently are prescribed to patients with early symptoms of dementia and to patients with severe or terminal illness and thus increasing the risk of dying [41, 56, 57]. Overdose with BZD rarely causes severe respiratory or cardiovascular depression and death [45]. However, in combined use with opioids or alcohol the risk of mortality increases [37, 58].

Use of opioids in older adults may be related to an altered state of consciousness [59], falls and fractures [59], over-sedation [59], respiratory depression [59], aspiration and pneumonia [60] leading to increased mortality [41]. In recent decades, concerns have been raised about misuse [40], overdose death [61, 62] and suicide death [63] among users of opioids in older adults.

There have also been some concerns regarding the combined use of alcohol and drugs with addiction potential in older adults [5, 36–38]. This combination can lead to serious side effects such as increased sedation, impaired coordination and breathing difficulties, which in turn can result in a greater risk of falls, accident and death [5, 36, 37]. To our knowledge, few studies have studied the mortality risk of the combined use of alcohol and drugs with addiction potential in older adults [64]. A study of adults (15–64 years) detected ethanol in 18.5% of opioid-related deaths, and patients whose death were related to opioids, were more likely to have a history of alcoholism [64].

In Norway, data on mortality related to use of alcohol and drugs with addiction potential in older adults is scarce [41, 65–67]. The aim of our study is therefore to investigate the association between drinking frequency, the use of prescribed drugs with addiction potential, the possible combination of frequent drinking and use of prescribed drugs with addiction potential and all-cause mortality as the outcome in older adults.

## Material and methods

### Study setting, data sources and participants

This study is based on data from the Nord-Trøndelag Health Study (HUNT) [68]. The HUNT study is a large population-based cohort study conducted in mid-Norway in Nord-Trøndelag County, which had a population of 128,694 in 2006 [69]. Nord-Trøndelag County is considered to be fairly representative of Norway regarding age and gender distribution, health status and mortality [70, 71]. Three waves of the HUNT study have been conducted thus far: HUNT1 (1984–1986), HUNT2 (1995–1997) and HUNT3 (2006–2008). This study is based on HUNT3 data, which was completed between October 2006 and June 2008 [69]. Every resident in the county aged 20 years and older was invited to participate and a total of 50,807 (54% of those invited) joined the HUNT3 study [69]. A non-participation study showed that non-participants in HUNT3 had lower socioeconomic status, higher prevalence of mental distress and chronic diseases and poorer health than those who participated [71]. Full details of the HUNT study have been provided elsewhere [71].

In HUNT3, the participation rate was highest in the 60–69 years (71.1%) and 70–79 years (66.8%) age groups. The participation rate was lower among the oldest age groups (41.6% in the 80–89 year-old age group and 17.2% in the 90+ age group) [67]. Poor physical health as a cause for non-participation in HUNT3 was in the age group 60–79 years almost 12% in women and 8% in men. This proportion increased to 24% and 20% among women and men 80 years or older, respectively [71].

This study relied on information from older adults who were 65 years or older when they participated in HUNT3. Of 12,361 participants ≥ 65 years, 816 (7%) were excluded because they had not answered the required question about drinking frequency. Thus, our study sample consisted of 11,545 individuals of the total sample of 12,361 (93%) individuals. Those not responding to the drinking frequency question (N = 816) were more often women, having higher age and a higher proportion had fewer years of education, no living spouse or partner, poorer health status, and self-reported chronic diseases. In addition, those not responding had higher mean score of HADS anxiety and HADS depression scales and used drugs with addiction potential more often than those responding to the question (p < 0.05).

Data on dispensed prescribed drugs with addiction potential were drawn from the Norwegian Prescription Database (NorPD) of the Norwegian Institute of Public Health from 2005 to 2009 and were linked to HUNT3 participants. The NorPD contains data on dispensed drugs for all citizens in Norway [72].

Data on death was drawn from the Norwegian Cause of Death Registry, which was linked to HUNT3 participants. Death information was obtained from 3 October 2006 to 31 December 2013. The Norwegian Cause of Death Registry covers all deaths in Norway [73].

### Measures

#### Alcohol consumption

HUNT3 included a question regarding how often participants had consumed alcohol in the last 12 months. The response options in HUNT3 were: 1 = 4–7 times a week, 2 = 2–3 times a week, 3 = about once a week, 4 = 2–3 times a month, 5 = about once a month, 6 = a few times a year, 7 = not at all in the last year and 8 = never drink alcohol.

In our study, participants who reported drinking alcohol a few times a year or more were defined as “current drinkers” (response categories 1–6) [21]. Drinking alcohol 4–7 times a week, 2–3 times a week, about once a week, 2–3 times a month and a few times a year were defined as drinking alcohol 4–7 days a week, 2–3 days a week, 1 day a week, 2–3 days a month and drinking occasionally (a few times a year), respectively. Frequent drinking was defined as drinking alcohol ≥ 4 days a week (response category 1).

Participants who reported that they had not consumed alcohol at all in the last year were defined as “non-drinkers last year” (response category 7), and participants who reported they never drink alcohol were defined as “never drinkers” (response category 8).

#### Drugs with addiction potential

Prescribed drugs with addiction potential in the HUNT3 sample were categorized according to the Anatomical Therapeutic Classification system (ATC) [74]. Drugs with addiction potential were defined as prescribed benzodiazepines (BZD), z-hypnotics or opioids. BZD were categorized under ATC codes N03AE (antiepileptic), N05BA (anxiolytic) and N05CD (hypnotic and sedative) [74]. Z-hypnotics were categorized under ATC code N05CF and opioids under ATC code N02A [74]. The use of prescribed drugs with addiction potential was defined as at least one prescription of BZD, z-hypnotics or opioids within one year for a minimum of two consecutive years (2005/2006, 2006/2007, 2007/2008 or 2008/2009) [75, 76]. The drugs were prescribed during the course of the HUNT3 survey.

#### Possible combination of use of alcohol and prescribed drugs with addiction potential

The possible combination of use of alcohol and prescribed drugs with addiction potential was defined as frequent drinking in HUNT3 and being prescribed drugs with addiction potential during the course of the HUNT3 survey.

#### Mortality

The main outcome was all-cause mortality. Follow-up continued until death or latest at 31 December 2013.

#### Independent variables

All socio-demographic, physical and mental health variables were self-reported and measured at the time of the HUNT3 survey completion (baseline assessment).

Socio-demographic variables. Our socio-demographic variables were gender, age at the time of survey completion, level of education (up to ten years of education, vocational and general education, college and university), urban versus rural living and marital status (living spouse or partner versus not). Age was dichotomized into two groups (65–74 years and 75 years or older) for the purpose of presenting separate models.

Smoking status. Smoking status was assessed using three categories: 1 = No, I have never smoked, 2 = No, I quit smoking, 3a) Yes, cigarettes occasionally, 3b) Yes, cigars/cigarillos, pipe occasionally, 3c) Yes, cigarettes daily or 3d) Yes, cigars/cigarillos, pipe daily. In our study response category 1 was defined as never smoked, response category 2 as former smoker and response categories from 3a to 3d as smoker.

Self-reported health status. Self-reported health status was assessed with the question: “How is your overall health for the time being?” The item had four response alternatives: very good, good, not so good and poor. We reversed the coding so that a higher score reflected a healthier state. As few individuals reported to have poor health status, the variable was dichotomized. Very good/good included response categories 3 and 4 and poor/not so good included response categories 1 and 2.

Medical diagnosis. In our study, diseases of the circulatory system were defined as self-reported myocardial infarction, heart failure, stroke or brain haemorrhage (yes/no). Diseases of the respiratory system were defined as self-reported chronic bronchitis, emphysema, chronic obstructive pulmonary disease or asthma (yes/no), and diseases of the musculoskeletal system were defined as self-reported arthritis, rheumatoid arthritis, Bechterew’s disease, osteoporosis, fibromyalgia, degenerative joint disease or osteoarthritis (yes/no). In addition, we had information about kidney disease (yes/no), diabetes (yes/no) and cancer (yes/no).

Anxiety and depression. Anxiety and depression were assessed by the self-reported instrument Hospital Anxiety and Depression scale (HADS) [77]. Anxiety symptoms (HAD-A) were assessed with 7 items, and depressive symptoms (HAD-D) were assessed with 7 items. Each item was scored 0–3, which produces a sum score from 0–21 on each subscale. In the analyses we used the continuous subscales (HAD-A and HAD-D) [78]. HADS has been validated in the general population and among older adults in Norway and has shown good psychometric properties [79, 80].

### Ethics and data protection

All HUNT3 participants signed an informed and written consent allowing use of their data for future medical research [69]. This consent included a provision allowing their data to be linked to other health records [70]. The Norwegian Institute of Public Health made the final link between HUNT3 [68], the NordPD [72] and the Norwegian Cause of Death registry [73]. To ensure anonymity according to Norwegian regulations governing the linkage of health records, all names and personal identification numbers were removed from the data files.

HUNT’s research is carried out in accordance with the guidelines of the Regional Committee of Medical Research Ethics (REC), the Norwegian Data Inspectorate Authority and applicable law [68]. REC (reference number 2014/1248), the Norwegian Social Science Data Services (project number 40081), the Norwegian Data Inspectorate Authority (reference number 14/01248-2EOL) and the Norwegian Institute of Public Health have all approved the present study.

### Statistical analyses

The data was analysed with SPSS version 24. Descriptive statistics were used to describe the baseline characteristics of the sample, both overall and according to mortality and drinking status. Categorical variables were analysed by Chi-Square test or by the Fisher’s Exact test (depending on expected values in the cells; i.e. less than 5). Age, HADS anxiety scale and HADS depression scale, as the only continuous descriptive variables, were not normally distributed, and analysed by the Mann-Whitney U test.

As we did not have information about the exact date of study entry in HUNT3 and the variability in timing of study entry was large (i.e. from 3 October 2006 to 25 June 2008), we were not able to estimate a valid time variable that could be used in Cox regression analyses. Moreover, we could not use age as a time variable in Cox regression analyses as we did not have information about the exact age at time of death [22]. Thus, we used binary logistic regression analyses (the Enter method) to investigate the association between the outcome measure all-cause mortality and three exposure variables: Model 1) drinking frequency (drinking occasionally (a few times a year) reference category), Model 2a) use of prescribed drugs with addiction potential (BZD, z-hypnotics or opioids) (versus not), Model 2b) use of prescribed BZD or z-hypnotics (versus not), Model 2c) use of prescribed BZD (versus not), Model 2d) use of prescribed z-hypnotics (versus not), Model 2e) use of prescribed opioids (versus not) and Model 3) the possible combination of frequent drinking (≥ 4 days a week) and use of prescribed drugs with addiction potential (versus no frequent drinking, no use of prescribed drugs with addiction potential or neither frequent drinking nor use of prescribed drugs with addiction potential).

Possible confounding was evaluated using Directed Acyclic Graphs (DAG) [81]. Gender [82–85], age [82–85], education [83, 85, 86], living area [85, 87], marital status [82, 85, 88], smoking [13, 66, 84, 89], overall health status [82, 83, 85] and physical health [82–84, 89, 90] and mental health [89–91] are associated with both the outcome measure all-cause mortality [13, 66, 84, 86–88, 91] and the exposure variables alcohol consumption [85, 90] and drugs with addiction potential [82, 83, 89]. These possible confounders were adjusted for in multivariate analyses, and therefore variables in the analyses were: gender (women reference category), age (continuous variable), level of education (up to ten years education reference category), living in urban versus rural areas, marital status (no living spouse or partner reference category), smoking status (never smoked reference category), overall health status (poor/not so good reference category), circulatory diseases (versus not), respiratory diseases (versus not), kidney disease (versus not), diabetes (versus not), cancer (versus not), musculoskeletal diseases (versus not), HADS anxiety scale (continuous variable) and HADS depression scale (continuous variable).

To reduce the chance of reverse causality [9, 65], we performed sensitivity analyses and repeated the analyses by excluding participants who reported to be never drinkers and non-drinkers in last year, those with self-reported medical diagnoses at baseline (circulatory diseases, respiratory diseases, kidney disease, diabetes, cancer and musculoskeletal diseases) and those who died within the first year after participation in HUNT3.

Statistical tests were carried out to assess for interactions between the exposure variables and gender and age. We found significant interactions between gender and drugs with addiction potential and between age and drugs with addiction potential. As women and the oldest age group versus men and the youngest age group may metabolise alcohol and drugs differently [5, 92, 93] and have different levels of alcohol consumption and use of drugs with addiction potential [1, 22, 83, 94], all multivariate analyses were stratified by gender and age groups (65–74 years versus 75 years or older). Unadjusted and adjusted analyses were presented with odds ratios (OR) and 95% confidence intervals (CI). Probability values below 0.05 were considered statistically significant.

Due to missing information on independent variables in women and men (see S1 and S2 Tables), the number of participants in the unadjusted and the adjusted analyses varied (see Table 1). Sensitivity analyses have been conducted in order to increase power and decrease missing by excluding three independent variables with the highest proportion of missing (education, HADS anxiety and HADS depression) in the adjusted analyses.

**Table 1:** Number of participants in unadjusted and adjusted multivariate analyses in women and men. The HUNT study 2006–08 (HUNT3).

	Unadjusted model: N	Adjusted model: N	Missing: (%)
Women overall ≥ 65 years	6084	3740	38.5
Women 65–74 years	3633	2406	33.8
Women ≥ 75 years	2451	1334	45.6
Men overall ≥ 65 years	5461	3522	35.5
Men 65–74 years	3455	2308	33.2
Men ≥ 75 years	2006	1214	39.5

## Results

Table 2 (women) and Table 3 (men) show the baseline characteristics of the study sample.

**Table 2:** Overall sample characteristics and according to mortality in older Norwegian women (≥ 65 years, N = 6,084). The HUNT Study 2006–08 (HUNT3).

		Overall	Alive	Dead	P-value
Overall	N (%)	6084 (100)	5332 (87.6)	752 (12.4)	
Age	Mean (SD)	73.9 (6.5)	73.2 (6.0)	79.1 (7.2)	
	Median (range)	72.8 (65–96.2)	72.1 (65–95.6)	80.1 (65–96.2)	< 0.001^a^
Age category					
65–74 years	N (%)* (%)**	3633 (59.7) (100)	3413 (64.0) (93.9)	220 (29.3) (6.1)	< 0.001^b^
≥ 75 years	N (%)* (%)**	2451 (40.3) (100)	1919 (36.0) (78.3)	532 (70.7) (21.7)	
Level of education^1^					
Up to ten years education	N (%)* (%)**	4602 (85.9) (100)	4031 (85.4) (87.6)	571 (90.1) (12.4)	0.006^b^
Vocational and general	N (%)* (%)**	116 (2.2) (100)	105 (2.2) (90.5)	11 (1.7) (9.5)	
College and university	N (%)* (%)**	637 (11.9) (100)	585 (12.4) (91.8)	52 (8.2) (8.2)	
Residence^1^					
Urban	N (%)* (%)**	3712 (61.6) (100)	3281 (62.1) (88.4)	431 (58.2) (11.6)	0.038^b^
Rural	N (%)* (%)**	2311 (38.4) (100)	2001 (37.9) (86.6)	310 (41.8) (13.4)	
Marital status^1^					
No living spouse or partner	N (%)* (%)**	2946 (48.4) (100)	2454 (46.0) (83.3)	492 (65.5) (16.7)	< 0.001^b^
Living spouse or partner	N (%)* (%)**	3136 (51.6) (100)	2877 (54.0) (91.7)	259 (34.5) (8.3)	
Smoking status^1^					
Never smoked	N (%)* (%)**	3055 (52.8) (100)	2686 (52.9) (87.9)	369 (51.9) (12.1)	0.003^b^
Former smoke r	N (%)* (%)**	1797 (31.1) (100)	1599 (31.5) (89.0)	198 (27.8) (11.0)	
Smoker	N (%)* (%)**	932 (16.1) (100)	788 (15.6) (84.5)	144 (20.3) (15.5)	
Overall health status^1^					
Poor/not so good	N (%)* (%)**	2452 (42.1) (100)	2017 (39.6) (82.3)	435 (59.9) (17.7)	< 0.001^b^
Good/very good	N (%)* (%)**	3373 (57.9) (100)	3082 (60.4) (91.4)	291 (40.1) (8.6)	
Circulatory diseases^1^^,^^2^	N (%)* (%)**	741 (12.2) (100)	549 (10.3) (74.1)	192 (25.5) (25.9)	< 0.001^b^
Respiratory diseases^1^^,^ ^3^	N (%)* (%)**	926 (15.2) (100)	778 (14.6) (84.0)	148 (19.7) (16.0)	< 0.001^b^
Kidney disease^1^	N (%)* (%)**	223 (3.7) (100)	179 (3.4) (80.3)	44 (5.9) (19.7)	0.001^b^
Diabetes ^1^	N (%)* (%)**	522 (8.6) (100)	418 (7.8) (80.0)	104 (13.9) (20.0)	< 0.001^b^
Cancer^1^	N (%)* (%)**	758 (12.5) (100)	597 (11.2) (78.8)	161 (21.4) (21.2)	< 0.001^b^
Musculoskeletal diseases^1^^,^ ^4^	N (%)* (%)**	3064 (53.4) (100)	2660 (62.7) (86.8)	404 (57.9) (13.2)	0.011^b^
HADS anxiety	Mean (SD)	4.2 (3.3)	4.2 (3.3)	4.2 (3.4)	
	Median (range)	4 (0–19)	4 (0–19)	4 (0–18)	0.430^a^
HADS depression	Mean (SD)	3.9 (2.9)	3.8 (2.9)	4.6 (3.0)	
	Median (range)	3 (0–18)	3 (0–18)	4 (0–14)	< 0.001^a^
Drinking frequency^5^					
Never	N (%)* (%)**	800 (13.1) (100)	668 (12.5) (83.5)	132 (17.6) (16.5)	< 0.001^b^
Not last year	N (%)* (%)**	676 (11.1) (100)	558 (10.5) (82.5)	118 (15.7) (17.5)	
Few times a year	N (%)* (%)**	1967 (32.3) (100)	1733 (32.5) (88.1)	234 (31.1) (11.9)	
Once a month	N (%)* (%)**	546 (9.0) (100)	488 (9.2) (89.4)	58 (7.8) (10.6)	
2–3 days a month	N (%)* (%)**	821 (13.5) (100)	735 (13.8) (89.5)	86 (11.4) (10.5)	
1 day a week	N (%)* (%)**	698 (11.5) (100)	621 (11.6) (89.0)	77 (10.2) (11.0)	
2–3 days a week	N (%)* (%)**	448 (7.4) (100)	411 (7.7) (91.7)	37 (4.9) (8.3)	
4–7 days a week	N (%)* (%)**	128 (2.1) (100)	118 (2.2) (92.2)	10 (1.3) (7.8)	
Drugs with addiction potential^6^					
BZD, z-hypnotics or opioids	N (%)* (%)**	2498 (41.1) (100)	2106 (39.5) (84.3)	392 (52.1) (15.7)	< 0.001^b^
BZD or z-hypnotics	N (%)* (%)**	2128 (35.0) (100)	1802 (33.8) (84.7)	326 (43.4) (15.3)	< 0.001^b^
BZD	N (%)* (%)**	977 (16.1) (100)	816 (15.3) (83.5)	161 (21.4) (16.5)	< 0.001^b^
Z-hypnotics	N (%)* (%)**	1542 (25.3) (100)	1309 (24.5) (84.9)	233 (31.0) (15.1)	< 0.011^b^
Opioids	N (%)* (%)**	892 (14.7) (100)	718 (13.5) (80.5)	174 (23.1) (19.5)	< 0.001^b^
Possible combination of alcohol consumption ≥ 4 days/week^5^ and use of prescribed drugs with addiction potential^6^	N (%)* (%)**	57 (0.9) (100)	54 (1.0) (94.7)	3 (0.4) (5.3)	0.102^b^

HADS = Hospital Anxiety and Depression Scale; BZD = benzodiazepines

*Column percent

**Row percent

^1^Number do not sum up to 6,084 because of missing information.

^2^Circulatory diseases defined as self-reported myocardial infarction, heart failure, stroke or brain haemorrhage.

^3^Respiratory diseases defined as self-reported asthma, chronic bronchitis, emphysema or chronic obstructive pulmonary disease.

^4^Musculoskeletal diseases defined as self-reported arthritis, rheumatoid arthritis, Bechterew’s disease, osteoporosis, fibromyalgia, degenerative joint disease or osteoarthritis.

^5^Self-reported alcohol consumption assessed among participants in HUNT3.

^6^Information about prescribed drugs with addiction potential among participants in HUNT3 (2006–08) was drawn from the Norwegian Prescription Database. Drugs with addiction potential were defined as at least one prescription of benzodiazepines, z-hypnotics or opioids in two consecutive years (2005/2006, 2006/2007, 2007/2008 or 2008/2009). Benzodiazepines defined by N03AE, N05BA and N05CD. Z-hypnotics defined by N05CF. Opioids defined by N02A.

^a^Significance testing with Mann-Whitney U test between alive and dead participants from 2006 to 2013 (all-cause mortality).

^b^Significance testing with Chi-square test between alive and dead participants from 2006 to 2013 (all-cause mortality).

**Table 3:** Overall sample characteristics and according to mortality in older Norwegian men (≥ 65 years, N = 5,461). The HUNT Study 2006–08 (HUNT3).

		Overall	Alive	Dead	P-value
Overall	N (%)	5461 (100)	4408 (80.7)	1053 (19.3)	
Age	Mean (SD)	73.5 (6.2)	72.4 (5.5)	77.8 (6.7)	
	Median (range)	72.4 (65–100.8)	71.4 (65–92.5)	78.4 (65–100.8)	< 0.001^a^
Age category					
65–74 years	N (%)* (%)**	3455 (63.3) (100)	3098 (70.3) (89.7)	357 (33.9) (10.3)	< 0.001^b^
≥ 75 years	N (%)* (%)**	2006 (36.7) (100)	1310 (29.7) (65.3)	696 (66.1) (34.7)	
Level of education^1^					
Up to ten years education	N (%)* (%)**	3819 (80.0) (100)	3061 (79.2) (80.2)	758 (83.2) (19.8)	0.014^b^
Vocational and general	N (%)* (%)**	139 (2.9) (100)	112 (2.9) (80.6)	27 (3.0) (19.4)	
College and university	N (%)* (%)**	817 (17.1) (100)	691 (17.9) (84.6)	126 (13.8) (15.4)	
Residence^1^					
Urban	N (%)* (%)**	3292 (60.9) (100)	2703 (62.0) (82.1)	589 (56.4) (17.9)	0.001^b^
Rural	N (%)* (%)**	2115 (39.1) (100)	1659 (38.0) (78.4)	456 (43.6) (21.6)	
Marital status^1^					
No living spouse or partner	N (%)* (%)**	1308 (24.0) (100)	1011 (22.9) (77.3)	297 (28.2) (22.7)	< 0.001^b^
Living spouse or partner	N (%)* (%)**	4151 (76.0) (100)	3396 (77.1) (81.8)	755 (71.8) (18.2)	
Smoking status^1^					
Never smoked	N (%)* (%)**	1469 (27.6) (100)	1277 (29.5) (86.9)	192 (19.1) (13.1)	< 0.001^b^
Former smoke r	N (%)* (%)**	2906 (54.6) (100)	2308 (53.5) (79.4)	598 (59.4) (20.6)	
Smoker	N (%)* (%)**	949 (17.8) (100)	733 (17.0) (77.2)	216 (21.5) (22.8)	
Overall health status^1^					
Poor/not so good	N (%)*(%)**	1899 (35.7) (100)	1332 (31.0) (70.1)	567 (55.5) (29.9)	< 0.001^b^
Good/very good	N (%)* (%)**	3414 (64.3) (100)	2960 (69.0) (86.7)	454 (44.5) (13.3)	
Circulatory diseases^1^^,^^2^	N (%)* (%)**	1236 (22.6) (100)	852 (19.3) (68.9)	384 (36.5) (31.1)	< 0.001^b^
Respiratory diseases^1^^,^ ^3^	N (%)* (%)**	833 (15.3) (100)	607 (13.8) (72.9)	226 (21.5) (27.1)	0.006^b^
Kidney disease^1^	N (%)* (%)**	250 (4.6) (100)	187 (4.2) (74.8)	63 (6.0) (25.2)	0.015^b^
Diabetes ^1^	N (%)* (%)**	554 (10.2) (100)	423 (9.6) (76.4)	131 (12.5) (23.6)	0.006^b^
Cancer^1^	N (%)* (%)**	700 (12.8) (100)	471 (10.7) (67.3)	229 (21.8) (32.7)	< 0.001^b^
Musculoskeletal diseases^1^^,^ ^4^	N (%)* (%)**	1374 (26.7) (100)	1057 (25.4) (76.9)	317 (32.2) (23.1)	< 0.001^b^
HADS anxiety	Mean (SD)	3.1 (2.7)	3.1 (2.7)	3.2 (2.8)	
	Median (range)	3 (0–18)	3 (0–18)	3 (0–17)	0.552^a^
HADS depression	Mean (SD)	4.1 (2.9)	4.0 (2.9)	4.7 (3.1)	
	Median (range)	4 (0–17)	4 (0–17)	4 (0–15)	< 0.001^a^
Drinking frequency^5^					
Never	N (%)* (%)**	234 (4.3) (100)	165 (3.7) (70.5)	69 (6.6) (29.5)	< 0.001^b^
Not last year	N (%)* (%)**	413 (7.6) (100)	285 (6.5) (69.0)	128 (12.2) (31.0)	
Few times a year	N (%)* (%)**	1245 (22.8) (100)	980 (22.2) (78.7)	265 (25.2) (21.3)	
Once a month	N (%)* (%)**	595 (10.9) (100)	476 (10.8) (80.0)	119 (11.4) (20.0)	
2–3 days a month	N (%)* (%)**	989 (18.1) (100)	811 (18.4) (82.0)	178 (16.9) (18.0)	
1 day a week	N (%)* (%)**	1007 (18.4) (100)	862 (19.6) (85.6)	145 (13.8) (14.4)	
2–3 days a week	N (%)* (%)**	739 (13.5) (100)	627 (14.2) (84.8)	112 (10.4) (15.2)	
4–7 days a week	N (%)* (%)**	239 (4.4) (100)	202 (4.6) (84.5)	37 (3.5) (15.5)	
Drugs with addiction potential^6^					
BZD, z-hypnotics or opioids	N (%)* (%)**	1243 (22.8) (100)	863 (19.6) (69.4)	380 (36.1) (30.6)	< 0.001^b^
BZD or z-hypnotics	N (%)* (%)**	902 (16.5) (100)	608 (13.8) (67.4)	294 (27.9) (32.6)	< 0.001^b^
BZD	N (%)* (%)**	388 (7.1) (100)	266 (6.0) (68.6)	122 (11.6) (31.4)	< 0.001^b^
Z-hypnotics	N (%)*(%)**	649 (11.9) (100)	430 (9.8) (66.3)	219 (20.8) (33.7)	< 0.001^b^
Opioids	N (%)* (%)**	541 (9.9) (100)	378 (8.6) (69.9)	163 (15.5) (30.1)	< 0.001^b^
Possible combination of alcohol consumption ≥ 4 days/week^5^ and use of prescribed drugs with addiction potential^6^	N (%)* (%)**	67 (1.2) (100)	53 (1.2) (79.1)	14 (1.3) (20.9)	0.736^b^

HADS = Hospital Anxiety and Depression Scale; BZD = benzodiazepines

*Column percent

**Row percent

^1^Number do not sum up to 5,461 because of missing information.

^2^Circulatory diseases defined as self-reported myocardial infarction, heart failure, stroke or brain haemorrhage.

^3^Respiratory diseases defined as self-reported asthma, chronic bronchitis, emphysema or chronic obstructive pulmonary disease.

^4^Musculoskeletal diseases defined as self-reported arthritis, rheumatoid arthritis, Bechterew’s disease, osteoporosis, fibromyalgia, degenerative joint disease or osteoarthritis.

^5^Self-reported alcohol consumption assessed among participants in HUNT3.

^6^Information about prescribed drugs with addiction potential among participants in HUNT3 (2006–08) was drawn from the Norwegian Prescription Database. Drugs with addiction potential were defined as at least one prescription of benzodiazepines, z-hypnotics or opioids in two consecutive years (2005/2006, 2006/2007, 2007/2008 or 2008/2009). Benzodiazepines defined by N03AE, N05BA and N05CD. Z-hypnotics defined by N05CF. Opioids defined by N02A.

^a^Significance testing with Mann-Whitney U test between alive and dead participants from 2006 to 2013 (all-cause mortality).

^b^Significance testing with Chi-square test between alive and dead participants from 2006 to 2013 (all-cause mortality).

Of all participating women, 800 (13.1%) were never drinkers, 676 (11.1%) were non-drinkers last year and 4,608 (75.8%) were current drinkers (drinking occasionally a few times a year or more). In men, 234 (4.3%) were never drinkers, 413 (7.6%) were non-drinkers last year and 4,814 (88.1%) were current drinkers. Women and men who reported to be never drinkers and non-drinkers last year were older and had a poorer health status than current drinkers (S3 to S6 Tables).

Table 2 (women) and Table 3 (men) show the prevalence of frequent drinking (≥ 4 days a week), use of drugs with addiction potential and the proportion of women and men who died within the period of follow-up (2006–2013).

Among both women and men who died, a higher proportion were never drinkers, non-drinkers in the last year and used prescribed drugs with addiction potential.

The association between drinking frequency, the use of prescribed drugs with addiction potential, the possible combination of frequent drinking and use of prescribed drugs with addiction potential and all-cause mortality as outcome are shown in Table 4 (women) and Table 5 (men).

**Table 4:** Association between drinking frequency, use of prescribed drugs with addiction potential and all-cause mortality among older Norwegian women (≥ 65 years) in unadjusted and adjusted logistic regression analyses. The HUNT Study 2006–08 (HUNT3).

	Unadjusted^a^ models	Adjusted^b^ models	Unadjusted^a^ models	Adjusted^b^ models	Unadjusted^a^ models	Adjusted^b^ models
	Overall ≥ 65 years	Overall ≥ 65 years	65–74 years	65–74 years	≥ 75 years	≥ 75 years
	N = 6084	N = 3740	N = 3633	N = 2406	N = 2451	N = 1334
	OR 95% CI	OR 95% CI	OR 95% CI	OR 95% CI	OR 95% CI	OR 95% CI
**Model 1:** Drinking frequency^1^						
Never	**1.46 (1.16–1.84)**	1.02 (0.71–1.46)	0.90 (0.54–1.51)	1.07 (0.55–2.09)	**1.43 (1.09–1.88)**	0.97 (0.63–1.50)
Not last year	**1.57 (1.23–1.99)**	0.86 (0.59–1.27)	1.07 (0.63–1.82)	0.60 (0.26–1.38)	**1.46 (1.10–1.93)**	0.89 (0.56–1.41)
Few times a year	1 (ref. category)	1 (ref. category)	1 (ref. category)	1 (ref. category)	1 (ref. category)	1 (ref. category)
Once a month	0.88 (0.65–1.19)	0.81 (0.52–1.26)	1.09 (0.67–1.76)	1.09 (0.59–2.03)	0.97 (0.64–1.45)	0.54 (0.28–1.03)
2–3 days a month	0.87 (0.67–1.13)	0.99 (0.68–1.44)	0.98 (0.64–1.50)	0.99 (0.57–1.74)	1.10 (0.77–1.55)	0.96 (0.57–1.61)
1 day a week	0.92 (0.70–1.21)	1.05 (0.71–1.57)	1.12 (0.73–1.71)	0.85 (0.47–1.54)	1.19 (0.81–1.71)	1.20 (0.69–2.08)
2–3 days a week	**0.67 (0.46–0.96)**	0.99 (0.60–1.62)	0.81 (0.47–1.40)	0.77 (0.37–1.60)	0.96 (0.57–1.61)	1.07 (0.53–2.18)
4–7 days a week	0.63 (0.32–1.21)	0.63 (0.23–1.74)	0.87 (0.34–2.21)	0.60 (0.14–2.62)	0.72 (0.27–1.89)	0.51 (0.12–2.15)
**Model 2a-2e:** Drugs with addiction potential^2^						
**Model 2a:** BZD, z- hypnotics or opioids	**1.67 (1.43–1.94)**	1.04 (0.82–1.33)	**1.45 (1.10–1.91)**	1.01 (0.68–1.51)	**1.34 (1.10–1.63)**	1.06 (0.77–1.45)
**Model 2b:** BZD or z- hypnotics	**1.50 (1.28–1.75)**	0.99 (0.78–1.27)	**1.35 (1.02–1.80)**	1.04 (0.69–1.56)	1.18 (0.97–1.43)	0.96 (0.70–1.31)
**Model 2c:** BZD	**1.50 (1.25–1.82)**	0.97 (0.72–1.31)	**1.73 (1.22–2.45)**	1.20 (0.71–2.02)	1.10 (0.87–1.38)	0.84 (0.58–1.23)
**Model 2d:** Z-hypnotics	**1.38 (1.17–1.63)**	1.09 (0.85–1.40)	1.20 (0.87–1.66)	0.98 (0.63–1.51)	1.13 (0.92–1.39)	1.16 (0.85–1.59)
**Model 2e:** Opioids	**1.94 (1.61–2.33)**	1.17 (0.87–1.59)	**1.97 (1.40–2.77)**	1.07 (0.63–1.82)	**1.62 (1.28–2.04)**	1.27 (0.87–1.85)
**Model 3:** Possible combination of alcohol consumption ≥ 4 days/week^1^ and use of prescribed drugs with addiction potential^2^	0.39 (0.12–1.26)	0.24 (0.03–1.85)	0.42 (0.06–3.05)	—	0.42 (0.10–1.83)	0.36 (0.04–3.12)

*Note*: Bold numbers indicate significant associations. OR = odds ratio; CI = confidence interval; BZD = benzodiazepines. Blank (—) indicates that we could not perform the analyses as none of the participants who possibly combined the use of alcohol ≥ 4 days a week and prescribed drugs with addiction potential died during the follow up period.

^1^Self-reported alcohol consumption assessed among participants in HUNT3.

^2^Information on prescribed drugs with addiction potential among participants in HUNT3 (2006–08) was drawn from the Norwegian Prescription Database. Drugs with addiction potential were defined as at least one prescription of BZD, z-hypnotics or opioids in one year for a minimum of two consecutive years (2005/2006, 2007/2008 or 2008/2009). BZD defined by N03AE, N05BA and N05CD. Z-hypnotics defined by N05CF. Opioids defined by N02A.

^a^Unadjusted binary logistic regression analysis. Dependent variable: All-cause mortality (2006–2013). Exposure variables: Model 1: Drinking frequency (drinking occasionally a few times a year reference category), Model 2a: BZD, z-hypnotics or opioids (no prescribed BZD, z-hypnotics or opioids reference category), Model 2b: BZD or z-hypnotics (no prescribed BZD or z-hypnotics reference category), Model 2c: BZD (no prescribed BZD reference category), Model 2d: z-hypnotics (no prescribed z-hypnotics reference category), Model 2e: opioids (no prescribed opioids reference category), Model 3: possible combination of alcohol consumption ≥ 4 days/week and use of prescribed drugs with addiction potential (BZD, z-hypnotics or opioids). Reference category: no alcohol consumption ≥ 4 days/week, no prescribed drugs with addiction potential, or neither alcohol consumption ≥ 4 days/week nor being prescribed drugs with addiction potential.

^b^Adjusted binary logistic regression analysis: Dependent variable: All-cause mortality (2006–2013). Exposure variables: The same as in the unadjusted analyses. Adjusted for gender (women reference category), age (continuous variable), level of education (up to ten year education reference category), living in urban versus rural areas, marital status (no living spouse or partner reference category), smoking status (never smoked reference category), overall health status (poor/not so good reference category), circulatory diseases (versus not), respiratory diseases (versus not), kidney disease (versus not), diabetes (versus not), cancer (versus not), musculoskeletal diseases (versus not), Hospital Anxiety and Depression Scale (HADS) anxiety scale (continuous variable) and HADS depression scale (continuous variable).

**Table 5:** Association between drinking frequency, use of prescribed drugs with addiction potential and all-cause mortality among older Norwegian men (≥ 65 years) in unadjusted and adjusted logistic regression analyses. The HUNT Study 2006–08 (HUNT3).

	Unadjusted^a^ models	Adjusted^b^ models	Unadjusted^a^ models	Adjusted^b^ models	Unadjusted^a^ models	Adjusted^b^ models
	Overall ≥ 65 years	Overall ≥ 65 years	65–74 years	65–74 years	≥ 75 years	≥ 75 years
	N = 5461	N = 3522	N = 3455	N = 2308	N = 2006	N = 1214
	OR 95% CI	OR 95% CI	OR 95% CI	OR 95% CI	OR 95% CI	OR 95% CI
**Model 1:** Drinking frequency^1^						
Never	**1.55 (1.13–2.11)**	**2.10 (1.33–3.43)**	1.33 (0.73–2.42)	1.34 (0.52–3.51)	**1.53 (1.03–2.26)**	**2.33 (1.33–4.08)**
Not last year	**1.66 (1.30–2.13)**	1.41 (0.97–2.07)	**1.90 (1.22–2.94)**	**2.33 (1.24–4.37)**	**1.45 (1.06–2.00)**	1.09 (0.68–1.75)
Few times a year	1 (ref. category)	1 (ref. category)	1 (ref. category)	1 (ref. category)	1 (ref. category)	1 (ref. category)
Once a month	0.93 (0.73–1.18)	1.16 (0.81–1.66)	1.02 (0.68–1.54)	1.10 (0.62–1.95)	1.06 (0.77–1.46)	1.27 (0.80–2.03)
2–3 days a month	0.81 (0.66–1.00)	1.26 (0.93–1.71)	0.93 (0.65–1.32)	1.31 (0.82–2.11)	1.10 (0.83–1.47)	1.31 (0.87–1.97)
1 day a week	**0.62 (0.50–0.78)**	0.94 (0.67–1.30)	0.79 (0.55–1.13)	1.10 (0.68–1.79)	0.83 (0.61–1.13)	0.78 (0.49–1.24)
2–3 days a week	**0.66 (0.52–0.84**)	1.02 (0.72–1.47)	0.81 (0.55–1.19)	1.03 (0.60–1.76)	1.03 (0.72–1.46)	1.08 (0.65–1.78)
4–7 days a week	**0.68 (0.47–0.99)**	1.10 (0.65–1.87)	0.83 (0.46–1.48)	1.10 (0.50–2.47)	0.87 (0.51–1.47)	1.10 (0.53–2.27)
**Model 2a-2e:** Drugs with addiction potential^2^						
**Model 2a:** BZD, z- hypnotics or opioids	**2.32 (2.00–2.68)**	**1.60 (1.28–2.00)**	**2.49 (1.96–3.16)**	**2.30 (1.65–3.20)**	**1.79 (1.47–2.18)**	1.27 (0.95–1.71)
**Model 2b:** BZD or z- hypnotics	**2.42 (2.06–2.84)**	**1.48 (1.15–1.89)**	**2.46 (1.89–3.20)**	**1.96 (1.33–2.88)**	**1.84 (1.48–2.28)**	1.28 (0.93–1.76)
**Model 2c:** BZD	**2.04 (1.63–2.56)**	1.41 (0.99–2.01)	**2.45 (1.72–3.50)**	**1.84 (1.07–3.16)**	**1.52 (1.11–2.07)**	1.14 (0.72–1.81)
**Model 2d:** Z-hypnotics	**2.43 (2.03–2.91)**	**1.57 (1.19–2.06)**	**2.21 (1.62–3.01)**	**1.90 (1.23–2.93)**	**1.89 (1.49–2.39)**	**1.50 (1.06–2.13)**
**Model 2e:** Opioids	**1.95 (1.60–2.38)**	**1.78 (1.34–2.37)**	**2.32 (1.71–3.14)**	**2.37 (1.59–3.54)**	**1.63 (1.23–2.15)**	1.42 (0.95–2.12)
**Model 3:** Possible combination of alcohol consumption ≥ 4 days/week^1^ and use of prescribed drugs with addiction potential^2^	1.11 (0.61–2.00)	1.53 (0.68–3.45)	1.59 (0.66–3.82)	2.16 (0.69–6.70)	0.75 (0.33–1.71)	1.24 (0.41–3.69)

*Note*: Bold numbers indicate significant associations. OR = odds ratio; CI = confidence interval; BZD = benzodiazepines

^1^Self-reported alcohol consumption assessed among participants in HUNT3.

^2^Information on prescribed drugs with addiction potential among participants in HUNT3 (2006–08) was drawn from the Norwegian Prescription Database. Drugs with addiction potential were defined as at least one prescription of BZD, z-hypnotics or opioids in one year for a minimum of two consecutive years (2005/2006, 2007/2008 or 2008/2009). BZD defined by N03AE, N05BA and N05CD. Z-hypnotics defined by N05CF. Opioids defined by N02A.

^a^Unadjusted binary logistic regression analysis. Dependent variable: All-cause mortality (2006–2013). Exposure variables: Model 1: Drinking frequency (drinking occasionally a few times a year reference category), Model 2a: BZD, z-hypnotics or opioids (no prescribed BZD, z-hypnotics or opioids reference category), Model 2b: BZD or z-hypnotics (no prescribed BZD or z-hypnotics reference category), Model 2c: BZD (no prescribed BZD reference category), Model 2d: z-hypnotics (no prescribed z-hypnotics reference category), Model 2e: opioids (no prescribed opioids reference category), Model 3: possible combination of alcohol consumption ≥ 4 days/week and use of prescribed drugs with addiction potential (BZD, z-hypnotics or opioids). Reference category: no alcohol consumption ≥ 4 days/week, no prescribed drugs with addiction potential, or neither alcohol consumption ≥ 4 days/week nor being prescribed drugs with addiction potential.

^b^Adjusted binary logistic regression analysis: Dependent variable: All-cause mortality (2006–2013). Exposure variables: The same as in the unadjusted analyses. Adjusted for gender (women reference category), age (continuous variable), level of education (up to ten year education reference category), living in urban versus rural areas, marital status (no living spouse or partner reference category), smoking status (never smoked reference category), overall health status (poor/not so good reference category), circulatory diseases (versus not), respiratory diseases (versus not), kidney disease (versus not), diabetes (versus not), cancer (versus not), musculoskeletal diseases (versus not), Hospital Anxiety and Depression Scale (HADS) anxiety scale (continuous variable) and HADS depression scale (continuous variable).

In adjusted logistic regression analyses among women, no associations were found between drinking frequency, including frequent drinking, and all-cause mortality compared to those who drank occasionally. Use of prescribed drugs with addiction potential and the possible combination of frequent drinking and use of prescribed drugs with addiction potential was not associated with mortality (Table 4).

In adjusted logistic regression analyses among men, frequent drinking was not associated with mortality compared to those who drank occasionally. The odds of mortality were more than two times higher in those aged 65–74 years who reported to be non-drinkers in the last year and among those who used prescribed drugs with addiction potential (Table 5). Furthermore, in men aged 75 years or older we found that those who never drank alcohol compared to occasionally drinking and those who used prescribed z-hypnotics had higher odds of mortality. No association was found between the possible combination of frequent drinking and use of prescribed drugs with addiction potential and mortality. However, men aged 65–74 years with this possible combination had more than two-fold higher odds of mortality, even though the association was not statistically significant with a wide confidence interval.

The adjusted sensitivity analyses (excluding never drinkers, non-drinkers in the last year, participants with self-reported medical diagnoses at baseline and participants who died within the first year after participation in HUNT3), showed no association between drinking frequency, including frequent drinking, and mortality in either women or men compared to those who drank occasionally (S9 and S10 Tables). Among men, we found no association between BZD and z-hypnotics and all-cause mortality. However, women and men aged 65–74 years who used prescribed opioids had more than four times higher odds of dying within the follow-up period.

We conducted adjusted sensitivity analyses by excluding three independent variables with the highest proportion of missing (education, HADS anxiety and HADS depression). The results were almost the same as in the main analyses, except for higher odds of mortality among women 75 years or older who used prescribed opioids (results not presented).

## Discussion

In this large Norwegian population-based HUNT study (HUNT3, 2006–08), frequent drinking (≥ 4 days a week) was not associated with all-cause mortality when compared to the reference category of drinking occasionally (a few times a year). Men aged 65–74 years who reported to be non-drinkers in the last year and men aged 75 years or older who reported to be never drinkers had higher odds of mortality compared to those who drank occasionally. Furthermore, in men, those who used prescribed drugs with addiction potential (BZD, z-hypnotics or opioids) had higher odds of mortality. No association was found between the possible combination of frequent drinking and use of drugs with addiction potential and mortality as outcome.

To avoid reverse causality we performed sensitivity analyses in women and men [9, 65] excluding both participants who reported to be never drinkers and non-drinkers in the last year, participants who died in the first year of follow-up and participants who reported medical diagnoses at study entry. The results from the adjusted sensitivity analyses were almost similar to the main results, except for higher odds of mortality among women using prescribed opioids, and no association between BZD and z-hypnotics and mortality among men. As in the main analyses, we did not find an association between frequent drinking and mortality in the sensitivity analyses. Moreover, we have performed adjusted sensitivity analyses by excluding three independent variables with the highest proportion of missing (education, HADS anxiety and HADS depression). The results from these analyses were also almost similar to the main results, with the exception of the association between use of opioids and mortality among women.

Several studies exploring the relation between heavy alcohol consumption using volume as exposure variable and all-cause mortality as the outcome have found an increased mortality risk in older adults [12, 13, 16, 25, 26], while others have not found this association [27, 29, 30]. A comparison between these studies and the present study using drinking frequency as the exposure variable is difficult. However, in line with our study, a longitudinal multicentre study performed in Europe and Russia found no association between frequent drinking and all-cause mortality [23].

The study results indicate that drinking alcohol four days a week or more by older Norwegian adults is not associated with increased mortality. This finding might be explained by the definition of frequent alcohol consumption (≥ 4 days a week) used in our study, which includes participants who exhibit both risky drinking (≥ 7 drinks a week) and non-risky drinking (< 7 drinks a week) [95]. Older adults who drink 4–7 drinks a week are considered to be low-to-moderate drinkers [16, 17, 96], and several studies have found a lower mortality risk in older adults with low-to-moderate alcohol consumption [11, 12, 29, 30, 33] or no association with survival [96]. This could indicate that low-to-moderate intake of alcohol is not unhealthy in old age. However, the fact that we found no association might also be because the study had a power problem, since few participating women and men from HUNT3 drank 4 days or more a week, and because of the low number of deaths among those with high drinking frequency. It is also possible that older adults with poor health might have stopped drinking alcohol [17], or those with heavy alcohol consumption may have died before study entry, leaving healthy survivors who are low-to-moderate drinkers with lower mortality risk [13]. Moreover, our study focused on the association between drinking frequency and all-cause mortality. However, alcohol consumption also has other impacts, such as on morbidity [21, 31] and hospitalization [97]. Thus, if we had used other health outcomes such as use of health care resources, we might have found an association between frequent drinking and the outcome.

In older adults, alcohol consumption might be a sign of good physical and mental health [4, 22]. This is partly supported by our finding where men who reported to be never drinkers and non-drinkers in the last year had poorer health than current drinkers, and men who abstained from alcohol at baseline had higher odds of mortality compared to those who drank occasionally. Other studies have also found that abstainers reported poorer health and died earlier than those who drank occasionally [20] or more regularly (low-to-moderate alcohol consumption) [13]. The higher odds of mortality among men who reported to be never drinkers and non-drinkers in the last year in our study might be due to health characteristics that differ between abstainers and current drinkers [22].

In the present study, the use of drugs with addiction potential (BZD, z-hypnotics or opioids) was associated with mortality in men. These findings are in line with previous studies [39–42, 61–63]. However, in older adults, associations between use of addictive psychotropic drugs (anxiolytics, sedative or hypnotics) and mortality have been inconsistent [39, 41–47]. Moreover, it is debated whether the association found between addictive psychotropic drugs and mortality is causal or not [41, 42, 45, 98]. Some authors have concluded that the increased mortality found with the use of addictive psychotropic is unlikely to be explained by confounding [42, 98], while other consider this association most likely to be due to confounding [41, 45]. In our study, we adjusted for several potential confounders measured at baseline such as health status, a number of different medical conditions, anxiety and depression, and we still found an association with mortality in men using drugs with addiction potential. Even so, the increased mortality among men who used these drugs might be due to a decrease in physical or mental health during follow-up. Men with serious health problems could be more likely to be prescribed drugs with addiction potential than healthy persons [41, 45, 99]. Consequently, it may be the participants’ health problems that contribute to adverse health outcomes, rather than the drug use. Thus, our finding with higher odds for mortality in men may be due to confounding and reverse causality [41, 43, 45, 65]. Furthermore, we did not find an association between use of prescribed drugs with addiction potential and mortality in women. The gender difference in mortality due to use of these drugs does not have an obvious explanation. However, it might be due to better health in women who use these drugs compared to men.

Older men with chronic conditions may be more likely than women to combine the use of alcohol and drugs with addiction potential [37]. However, in a previous study we found that a higher proportion of older women (39.6%) than men (22.8%) who were regular drinkers (alcohol consumption ≥ 1 day a week) also used drugs with addiction potential [38]. Thus, it seems that both older women and men may use alcohol in addition to drugs with addiction potential [38]. Nevertheless, we did not find an association in men or women between a possible combination of frequent drinking and use of prescribed drugs with addiction potential and mortality. This might be explained by the limited power of our study, as very few participants in HUNT3 presented with a possible combination of frequent drinking and use of prescribed drug with addiction potential. The wide confidence intervals in the adjusted analyses also indicate limited power. However, a two-fold higher odds of mortality among men aged 65–74 years with a possible combination of frequent drinking and use of prescribed drugs with addiction potential might be of clinical importance, even though the association was not statistically significant and had a wide confidence interval. Any conclusion regarding this association from our study need to be replicated in a new study with a larger number of older women and men with a possible combination of alcohol and drugs with addiction potential.

### Strengths and limitations

Our study has several strengths. Firstly, the large sample size in this population-based study made it possible to control for a range of variables that could confound the association under study. Secondly, data from the Norwegian Prescription Database [72] were used. Thus, information was available about all dispensed BZD, z-hypnotics and opioids prescriptions and prevents selection bias and recall bias of drug use [100]. Even so, we do not know if the participants took the dispensed drugs or what the indications for use of the drugs were [100]. Using the definition of at least one prescription in two consecutive years minimized the misclassification of use. A second prescription strongly suggests that the first had been filled and taken [101]. Thirdly, information about death was obtained from the Norwegian Causes of Death Registry [73]. The quality of national registries in Norway is high, and the validity of all-cause mortality as the outcome is considered to be very high [102].

However, our study has some limitations. Firstly, as we did not have information about the date of study entry and the exact age at time of death, we used logistic regression analyses to investigate the association between drinking frequency, use of drugs with addiction potential and the outcome all-cause mortality. However, we do not believe that the results would have changed much with the use of Cox regression analysis. A previous study investigating the relationship between alcohol consumption and all-cause mortality used both logistic regression analysis and Cox regression analysis and the results of both methods were practically identical [103].

Secondly, all data were collected at baseline, and we lack information about consumed alcohol volume. For example, several assessments of alcohol consumption, including the consumed volume, could have given us a better estimate of the exposure variable and the stability of the alcohol consumption over time [18, 22]. This may be of importance in older adults since alcohol consumption in general declines by increasing age [25] and some may have quit drinking during follow-up [16, 17].

Thirdly, the participation rate in HUNT3 decreased considerably with increasing age [69]. This limited statistical power in the oldest age group. Furthermore, a non-participant study showed that a relatively high proportion of non-participating older adults (≥ 60 years) reported to have too poor physical health to attend the HUNT3-study, and non-participants had higher mortality, higher prevalence of several chronic diseases and mental distress compared to participating older adults [71]. This might decrease the prevalence of frequent alcohol consumption. Even so, previous non-participant studies from Norwegian health surveys (the HUNT2 study and the Hordaland Health Study) have found that non-participation is higher among individuals with mental disorders and substance use disorders (i.e. use of alcohol, opioids, sedatives, hypnotics or anxiolytics) [104, 105]. Thus, older adults with alcohol disorders and/or substance disorders due to use of drugs with addition potential may be less likely to participate in the present study.

Fourthly, the proportion of missing data was quite high in some of the independent variables, and it is likely that those not responding was older and had poorer physical health. Those not answering the drinking frequency question was also older and a higher proportion had chronic diseases and used drugs with addiction potential. Therefore, the prevalence of drugs with addiction potential is most likely underestimated in our study. Moreover, older adults who are not drinking alcohol may not respond to the drinking frequency question as they may assume that this question is not relevant for them and thus affecting the prevalence of drinking frequency. Therefore, missing of this item is less likely to be at random.

Fifthly, our follow-up period was until five year, and relatively short [24]. A longer follow-up period would likely lead to larger numbers of deaths, which in turn could give us more precise estimates [24]. However, a short follow-up period ensures less change in alcohol use from baseline.

Lastly, like previous studies investigating the association between alcohol consumption [20, 21], drugs with addiction potential [39, 40] and all-cause mortality, the present study is an observational study, and our findings do not prove causality [26, 41]. Moreover, even in this relatively large cohort, we encountered power problems due to having few participants with high drinking frequency, and this may have influenced our results.

## Conclusion

Frequent drinking (≥ 4 days a week) compared to occasionally drinking (a few times a year) and the possible combination of frequent drinking and use of prescribed drugs with addiction potential was not associated with all-cause mortality in older women and men. Men who reported to be never drinkers and non-drinkers in the last year had higher odds of mortality compared to those drinking occasionally. Use of prescribed drugs with addiction potential was associated with higher odds of mortality in men. This finding should lead to more caution in prescribing drugs with addiction potential to this group. Aside from this, findings from the present epidemiological study do not have obvious clinical implications. More research is needed to shed light on the research questions raised in our study, questions which remain of great importance both from a clinical and public health perspective.

## Supporting information

S1 Table Missing in independent variables among women ≥ 65 years who had answered the alcohol frequency question (N = 6084). The HUNT Study 2006–08 (HUNT3).
(DOCX)

S2 Table Missing in independent variables among men ≥ 65 years who had answered the alcohol frequency question (N = 5461). The HUNT Study 2006–08 (HUNT3).
(DOCX)

S3 Table Overall sample characteristics and according to drinking status (never drinkers versus current drinkers) in older Norwegian women (≥ 65 years, N = 5,408). Non-drinkers in the last year excluded. The HUNT Study 2006–08 (HUNT3).
(DOCX)

S4 Table Overall sample characteristics and according to drinking status (never drinkers versus current drinkers) in older Norwegian men (≥ 65 years, N = 5,048). Non-drinkers last year excluded. The HUNT Study 2006–08 (HUNT3).
(DOCX)

S5 Table Overall sample characteristics and according to drinking status (non-drinkers last year versus current drinkers) in older Norwegian women (≥ 65 years, N = 5,284). Never drinkers excluded. The HUNT Study 2006–08 (HUNT3).
(DOCX)

S6 Table Overall sample characteristics and according to drinking status (non-drinkers last year versus current drinkers) in older Norwegian men (≥ 65 years, N = 5,227). Never drinkers excluded. The HUNT Study 2006–08 (HUNT3).
(DOCX)

S7 Table Sensitivity analyses in older Norwegian women (≥ 65 years). Never drinkers, non-drinkers last year, those with medical diagnoses at baseline^a^ and those who died within the first year after participation in HUNT3 all excluded. Overall sample characteristics and according to mortality (N = 1,337). The HUNT Study 2006–08 (HUNT3).
(DOCX)

S8 Table Sensitivity analyses in older Norwegian men (≥ 65 years). Never drinkers, non-drinkers last year, those with medical diagnoses at baseline^a^ and those who died within the first year after participation in HUNT3 all excluded. Overall sample characteristics and according to mortality (N = 1,783). The HUNT Study 2006–08 (HUNT3).
(DOCX)

S9 Table Sensitivity analyses in older Norwegian women (≥ 65 years). Never drinkers, non-drinkers last year, those with medical diagnoses at baseline^a^ and those who died within the first year after participation in HUNT3 all excluded. Association between drinking frequency, use of prescribed drugs with addiction potential and all-cause mortality in unadjusted and adjusted logistic regression analyses. The HUNT Study 2006–08 (HUNT3).
(DOCX)

S10 Table Sensitivity analyses in older Norwegian men (≥ 65 years). Never drinkers, non-drinkers last year, those with medical diagnoses at baseline^a^ and those who died within the first year after participation in HUNT3 all excluded. Association between drinking frequency, use of prescribed drugs with addiction potential and all-cause mortality in unadjusted and adjusted logistic regression analyses. The HUNT Study 2006–08 (HUNT3).
(DOCX)
