# Supplementary material for: Mortality in older adults with frequent alcohol consumption and use of drugs with addiction potential – The Nord Trøndelag Health Study 2006-2008 (HUNT3), Norway, a population-based study
Source: PLoS One. 2019 Apr 16;14(4):e0214813. doi: 10.1371/journal.pone.0214813 (PMC6467384; doi:10.1371/journal.pone.0214813)
Supplement: S1 Table — The HUNT Study 2006–08 (HUNT3). (DOCX) [file pone.0214813.s001.docx]

**S1 Table: Missing in independent variables among women ≥ 65 years who had answered the alcohol frequency question (N = 6084). The HUNT Study 2006-08 (HUNT3)**

| **Independent variables** | **Valid: N (%)** | **Missing: N %)** |
| --- | --- | --- |
| Age | 6084 (100) | 0 (0) |
| Education | 5355 (88) | 729 (12) |
| Residence: urban/rural | 6023 (99) | 61 (1.0) |
| Marital status | 6082 (100) | 2 (0) |
| Smoking status | 5784 (95.1) | 300 (4.9) |
| Health status | 5825 (95.7) | 259 (4.3) |
| Circulatory diseases | 6084 (100) | 0 (0) |
| Respiratory diseases | 6081 (100) | 3 (0) |
| Kidney disease | 6082 (100) | 2 (0) |
| Diabetes | 6081 (100) | 3 (0) |
| Cancer | 6082 (100) | 2 (0) |
| Musculoskeletal diseases | 5743 (94.4) | 341 (5.6) |
| HADS anxiety | 4927 (81) | 1157 (19) |
| HADS depression | 5089 (83.6) | 995 (16.4) |
| Drugs with addiction potential | 6084 (100) | 0 (0) |

HADS = Hospital Anxiety and Depression Scale
