# Supplementary material for: Mortality in older adults with frequent alcohol consumption and use of drugs with addiction potential – The Nord Trøndelag Health Study 2006-2008 (HUNT3), Norway, a population-based study
Source: PLoS One. 2019 Apr 16;14(4):e0214813. doi: 10.1371/journal.pone.0214813 (PMC6467384; doi:10.1371/journal.pone.0214813)
Supplement: S2 Table — The HUNT Study 2006–08 (HUNT3). (DOCX) [file pone.0214813.s002.docx]

**S2 Table: Missing in independent variables among men ≥ 65 years who had answered the alcohol frequency question (N = 5461). The HUNT Study 2006-08 (HUNT3).**

| **Independent variables** | **Valid: N (%)** | **Missing: N %)** |
| --- | --- | --- |
| Age | 5461 (100) | 0 (0) |
| Education | 4775 (87.4) | 686 (12.6) |
| Residence: urban/rural | 5407 (99) | 54 (1) |
| Marital status | 5459 (100) | 2 (0) |
| Smoking status | 5324 (97.5) | 137 (2.5) |
| Health status | 5313 (97.3) | 148 (2.7) |
| Circulatory diseases | 5460 (100) | 1 (0) |
| Respiratory diseases | 5455 (99.9) | 6 (0.1) |
| Kidney disease | 5458 (99.9) | 3 (0.1) |
| Diabetes | 5458 (99.9) | 3 (0.1) |
| Cancer | 5458 (99.9) | 3 (0.1) |
| Musculoskeletal diseases | 5147 (94.3) | 314 (5.7) |
| HADS anxiety | 4509 (82.6) | 952 (17.4) |
| HADS depression | 4617 (84.5) | 844 (15.5) |
| Drugs with addiction potential | 5461 (100) | 0 (0) |

HADS = Hospital Anxiety and Depression Scale
