# Supplementary material for: Mortality in older adults with frequent alcohol consumption and use of drugs with addiction potential – The Nord Trøndelag Health Study 2006-2008 (HUNT3), Norway, a population-based study
Source: PLoS One. 2019 Apr 16;14(4):e0214813. doi: 10.1371/journal.pone.0214813 (PMC6467384; doi:10.1371/journal.pone.0214813)
Supplement: S3 Table — Non-drinkers in the last year excluded. The HUNT Study 2006–08 (HUNT3). (DOCX) [file pone.0214813.s003.docx]

**S3 Table: Overall sample characteristics and according to drinking status (never drinkers versus current drinkers) in older Norwegian women (≥ 65 years, N = 5,408). Non-drinkers in the last year excluded. The HUNT Study 2006-08 (HUNT3)**

**Overall** **Never drinkers^a^** **Current drinkers^a^ p-value**

Overall N (%) 5408 (100) 800 (14.8) 4608 (85.2)

Age Mean (SD) 73.6 (6.4) 76.4 (6.8) 73.2 (6.2)

Median (range) 72.5 (65-96.2) 75.9 (65-94.4) 72 (65-96.2) < 0.001^b^

Age category

65-74 years N (%)* (%)** 3340 (61.8) (100) 362 (45.3) (10.8) 2978 (64.6) (89.2) < 0.001^c^

≥ 75 years N (%)* (%)** 2068 (38.2) (100) 438 (54.7) (21.2) 1630 (35.4) (78.8)

Level of education^1^

Up to ten years education N (%)* (%)** 4085 (85.2) (100) 591 (87.5) (14.5) 3494 (84.8) (85.5) 0.111^c^

Vocational and general N (%)* (%)** 113 (2.4) (100) 10 (1.5) (8.8) 103 (2.5) (91.2)

College and university N (%)* (%)** 595 (12.4) (100) 74 (11.0) (12.4) 521 (12.7) (87.6)

Residence^1^

Urban N (%)* (%)** 3345 (62.5) (100) 459 (57.5) (13.7) 2886 (63.3) (86.3) 0.002^c^

Rural N (%)* (%)** 2009 (37.5) (100) 339 (42.5) (16.9) 1670 (36.7) (83.1)

Marital status^1^

No living spouse or partner N (%)* (%)** 2556 (47.3) (100) 450 (56.3) (17.6) 2106 (45.7) (82.4) < 0.001^c^

Living spouse or partner N (%)* (%)** 2851 (52.7) (100) 350 (43.7) (12.3) 2501 (54.3) (87.7)

Smoking status^1^

Never smoked N (%)* (%)** 2713 (52.6) (100) 648 (84.4) (23.9) 2065 (47.1) (76.1) < 0.001^c^

Former smoker N (%)* (%)** 1601 (31.1) (100) 75 (9.8) (4.7) 1526 (34.8) (95.3)

Smoker N (%)* (%)** 840 (16.3) (100) 45 (5.8) (5.4) 795 (18.1) (94.6)

Overall health status^1^

Poor/not so good N (%)* (%)** 2111 (40.8) (100) 393 (51.2) (18.6) 1718 (38.9) (81.4) < 0.001^c^

Good/very good N (%)* (%)** 3069 (59.2) (100) 375 (48.8) (12.2) 2694 (61.1) (87.8)

Circulatory diseases^1, 2^ N (%)* (%)** 623 (11.5) (100) 117 (14.6) (18.8) 506 (11.0) (81.2) 0.003^c^

Respiratory diseases^1, 3^ N (%)* (%)** 810 (15.0) (100) 107 (13.4) (13.2) 703 (15.3) (86.8) 0.172^c^

Kidney disease^1^ N (%)* (%)** 192 (3.6) (100) 34 (4.3) (17.7) 158 (3.4) (82.3) 0.247^c^

Diabetes ^1^ N (%)* (%)** 447 (8.3) (100) 112 (14.0) (25.1) 335 (7.3) (74.9) < 0.001^c^

Cancer^1^ N (%)* (%)** 673 (12.4) (100) 108 (13.5) (16.1) 565 (12.3) (83.9) 0.321^c^

Musculoskeletal diseases^1, 4^ N (%)* (%)** 2697 (52.9) (100) 416 (56.4) (15.4) 2281 (52.3) (84.6) 0.039^c^

**Overall** **Never drinkers^a^** **Current drinkers^a^ p-value**

HADS anxiety Mean (SD) 4.2 (3.3) 4.0 (3.5) 4.2 (3.2)

Median (range) 4 (0-19) 3 (0-17) 4 (0-19) 0.011^b^

HADS depression Mean (SD) 3.8 (2.9) 4.1 (3.1) 3.8 (2.8)

Median (range) 3 (0-18) 4 (0-14) 3 (0-18) 0.111^b^

Drugs with addiction potential^5^

BZD, z-hypnotics or opioids N (%)* (%)** 2160 (39.9) (100) 324 (40.5) (15.0) 1836 (39.8) (85.0) 0.726^c^

BZD or z-hypnotics N (%)* (%)** 1837 (34.0) (100) 276 (34.5) (15.0) 1561 (33.9) (85.0) 0.731^c^

BZD N (%)* (%)** 824 (15.2) (100) 156 (19.5) (18.9) 668 (14.5) (81.1) < 0.001^c^

Z-hypnotics N (%)* (%)** 1349 (24.9) (100) 179 (22.4) (13.3) 1170 (25.4) (86.7) 0.069^c^

Opioids N (%)* (%)** 759 (14.0) (100) 126 (15.8) (16.6) 633 (13.7) (83.4) 0.130^c^

HADS = Hospital Anxiety and Depression Scale; BZD = benzodiazepines

*Column percent

**Row percent

^1^Number do not sum up to 5,408 because of missing information.

^2^Circulatory diseases defined as self-reported myocardial infarction, heart failure, stroke or brain haemorrhage.

^3^Respiratory diseases defined as self-reported asthma, chronic bronchitis, emphysema or chronic obstructive pulmonary disease.

^4^Musculoskeletal diseases defined as self-reported arthritis, rheumatoid arthritis, Bechterew’s disease, osteoporosis, fibromyalgia, degenerative joint disease or osteoarthritis.

^5^Information about prescribed drugs with addiction potential among participants in HUNT3 (2006-08) was drawn from the Norwegian Prescription Database. Drugs with addiction potential were defined as at least one prescription of benzodiazepines, z-hypnotics or opioids in two consecutive years (2005/2006, 2006/2007, 2007/2008 or 2008/2009). Benzodiazepines defined by N03AE, N05BA and N05CD. Z-hypnotics defined by N05CF. Opioids defined by N02A.

^a^Self-reported alcohol consumption assessed among participants in HUNT3. Current drinkers defined as drinking at least a few times a year.

^b^Significance testing with Mann-Whitney U test between never drinkers and current drinkers.

^c^Significance testing with Chi-square test between never drinkers and current drinkers.
