# Supplementary material for: Mortality in older adults with frequent alcohol consumption and use of drugs with addiction potential – The Nord Trøndelag Health Study 2006-2008 (HUNT3), Norway, a population-based study
Source: PLoS One. 2019 Apr 16;14(4):e0214813. doi: 10.1371/journal.pone.0214813 (PMC6467384; doi:10.1371/journal.pone.0214813)
Supplement: S4 Table — Non-drinkers last year excluded. The HUNT Study 2006–08 (HUNT3). (DOCX) [file pone.0214813.s004.docx]

**S4 Table: Overall sample characteristics and according to drinking status (never drinkers versus current drinkers) in older Norwegian men (≥ 65 years, N = 5,048). Non-drinkers last year excluded. The HUNT Study 2006-08 (HUNT3)**

**Overall** **Never drinkers^a^** **Current drinkers^a^ p-value**

Overall N (%) 5048 (100) 234 (4.6) 4814 (95.4)

Age Mean (SD) 73.3 (6.0) 76.0 (6.5) 73.1 (6.0)

Median (range) 72.2 (65-97.7) 75.4 (65.1-93.0) 72.1 (65-97.7) < 0.001^b^

Age category

65-74 years N (%)* (%)** 3261 (64.6) (100) 109 (46.6) (3.3) 3152 (65.5) (96.7) < 0.001^c^

≥ 75 years N (%)* (%)** 1787 (35.4) (100) 125 (53.4) (7.0) 1662 (34.5) (93.0)

Level of education^1^

Up to ten years education N (%)* (%)** 3521 (79.5) (100) 162 (77.8) (4.6) 3359 (79.5) (95.4) 0.097^c^

Vocational and general N (%)* (%)** 132 (3.0) (100) 2 (1.0) (1.5) 130 (3.1) (98.5)

College and university N (%)* (%)** 778 (17.5) (100) 44 (21.2) (5.7) 734 (17.4) (94.3)

Residence^1^

Urban N (%)* (%)** 3068 (61.4) (100) 139 (59.4) (4.5) 2929 (61.5) (95.5) 0.518^c^

Rural N (%)* (%)** 1928 (38.6) (100) 95 (40.6) (4.9) 1833 (38.5) (95.1)

Marital status^1^

No living spouse or partner N (%)* (%)** 1186 (23.5) (100) 62 (26.5) (5.2) 1124 (23.4) (94.8) 0.269^c^

Living spouse or partner N (%)* (%)** 3860 (76.5) (100) 172 (73.5) (4.5) 3688 (76.6) (95.5)

Smoking status^1^

Never smoked N (%)* (%)** 1359 (27.6) (100) 160 (71.7) (11.8) 1199 (25.5) (88.2) < 0.001^c^

Former smoker N (%)* (%)** 2676 (54.3) (100) 47 (21.1) (1.8) 2629 (55.9) (98.2)

Smoker N (%)* (%)** 890 (18.1) (100) 16 (7.2) (1.8) 874 (18.6) (98.2)

Overall health status^1^

Poor/not so good N (%)* (%)** 1699 (34.6) (100) 93 (40.8) (5.5) 1606 (34.3) (94.5) 0.043^c^

Good/very good N (%)* (%)** 3215 (65.4) (100) 135 (59.2) (4.2) 3080 (65.7) (95.8)

Circulatory diseases^1, 2^ N (%)* (%)** 1108 (22.0) (100) 55 (23.5) (5.0) 1053 (21.9) (95.0) 0.557^c^

Respiratory diseases^1, 3^ N (%)* (%)** 763 (15.1) (100) 33 (14.1) (4.3) 730 (15.2) (95.7) 0.652^c^

Kidney disease^1^ N (%)* (%)** 227 (4.5) (100) 13 (5.6) (5.7) 214 (4.4) (94.3) 0.415^c^

Diabetes ^1^ N (%)* (%)** 498 (9.9) (100) 31 (13.2) (6.2) 467 (9.7) (93.8) 0.076^c^

Cancer^1^ N (%)* (%)** 636 (12.6) (100) 26 (11.1) (4.1) 610 (12.7) (95.9) 0.481^c^

Musculoskeletal diseases^1, 4^ N (%)* (%)** 1243 (26.1) (100) 64 (28.8) (5.1) 1179 (26.0) (94.9) 0.344^c^

**Overall** **Never drinkers^a^** **Current drinkers^a^ p-value**

HADS anxiety Mean (SD) 3.1 (2.7) 2.6 (2.7) 3.1 (2.7)

Median (range) 2 (0-17) 2 (0-13) 3 (0-17) 0.002^b^

HADS depression Mean (SD) 4.1 (2.9) 3.7 (2.8) 4.1 (2.9)

Median (range) 4 (0-17) 3 (0-15) 4 (0-17) 0.087^b^

Drugs with addiction potential^5^

BZD, z-hypnotics or opioids N (%)* (%)** 1122 (22.2) (100) 37 (15.8) (3.3) 1085 (22.5) (96.7) 0.016^c^

BZD or z-hypnotics N (%)* (%)** 809 (16.0) (100) 29 (12.4) (3.6) 780 (16.2) (96.4) 0.121^c^

BZD N (%)* (%)** 335 (6.6) (100) 12 (5.1) (3.6) 323 (6.7) (96.4) 0.343^c^

Z-hypnotics N (%)* (%)** 591 (11.7) (100) 19 (8.1) (3.2) 572 (11.9) (96.8) 0.080^c^

Opioids N (%)* (%)** 484 (9.6) (100) 11 (4.7) (2.3) 473 (9.8) (97.7) 0.009^c^

HADS = Hospital Anxiety and Depression Scale; BZD = benzodiazepines

*Column percent

**Row percent

^1^Number do not sum up to 5,048 because of missing information.

^2^Circulatory diseases defined as self-reported myocardial infarction, heart failure, stroke or brain haemorrhage.

^3^Respiratory diseases defined as self-reported asthma, chronic bronchitis, emphysema or chronic obstructive pulmonary disease.

^4^Musculoskeletal diseases defined as self-reported arthritis, rheumatoid arthritis, Bechterew’s disease, osteoporosis, fibromyalgia, degenerative joint disease or osteoarthritis.

^5^Information about prescribed drugs with addiction potential among participants in HUNT3 (2006-08) was drawn from the Norwegian Prescription Database. Drugs with addiction potential were defined as at least one prescription of benzodiazepines, z-hypnotics or opioids in two consecutive years (2005/2006, 2006/2007, 2007/2008 or 2008/2009). Benzodiazepines defined by N03AE, N05BA and N05CD. Z-hypnotics defined by N05CF. Opioids defined by N02A.

^a^Self-reported alcohol consumption assessed among participants in HUNT3. Current drinkers defined as drinking at least a few times a year.

^b^Significance testing with Mann-Whitney U test between never drinkers and current drinkers.

^c^Significance testing with Chi-square test between never drinkers and current drinkers.
