# Supplementary material for: Mortality in older adults with frequent alcohol consumption and use of drugs with addiction potential – The Nord Trøndelag Health Study 2006-2008 (HUNT3), Norway, a population-based study
Source: PLoS One. 2019 Apr 16;14(4):e0214813. doi: 10.1371/journal.pone.0214813 (PMC6467384; doi:10.1371/journal.pone.0214813)
Supplement: S5 Table — Never drinkers excluded. The HUNT Study 2006–08 (HUNT3). (DOCX) [file pone.0214813.s005.docx]

**S5 Table: Overall sample characteristics and according to drinking status (non-drinkers last year versus current drinkers) in older Norwegian women (≥ 65 years, N = 5,284). Never drinkers excluded. The HUNT Study 2006-08 (HUNT3)**

**Overall** **Non-drinkers**  **Current drinkers^a^ p-value**

**last year^a^**

Overall N (%) 5284 (100) 676 (12.8) 4608 (87.2)

Age Mean (SD) 73.6 (6.3) 76.4 (6.8) 73.2 (6.2)

Median (range) 72.4 (65-96.2) 76.2 (65-95.6) 72 (65-96.2) < 0.001^b^

Age category

65-74 years N (%)* (%)** 3271 (61.9) (100) 293 (43.3) (9.0) 2978 (64.6) (91.0) < 0.001^c^

≥ 75 years N (%)* (%)** 2013 (38.1) (100) 383 (56.7) (19.0) 1630 (35.4) (81.0)

Level of education^1^

Up to ten years education N (%)* (%)** 4011 (85.7) (100) 517 (92.0) (12.9) 3494 (84.8) (87.1) < 0.001^c^

Vocational and general N (%)* (%)** 106 (2.3) (100) 3 (0.5) ((2.8) 103 (2.5) (97.2)

College and university N (%)* (%)** 563 (12.0) (100) 42 (7.5) (7.5) 521 (12.7) (92.5)

Residence^1^

Urban N (%)* (%)** 3253 (62.3) (100) 367 (54.9) (11.3) 2886 (63.3) (88.7) < 0.001^c^

Rural N (%)* (%)** 1972 (37.7) (100) 302 (45.1) (15.3) 1670 (36.7) (84.7)

Marital status^1^

No living spouse or partner N (%)* (%)** 2496 (47.3) (100) 390 (57.8) (15.6) 2106 (45.7) (84.4) < 0.001^c^

Living spouse or partner N (%)* (%)** 2786 (52.7) (100) 285 (42.2) (10.2) 2501 (54.3) (89.8)

Smoking status^1^

Never smoked N (%)* (%)** 2407 (48.0) (100) 342 (54.3) (14.2) 2065 (47.1) (85.8) 0.002^c^

Former smoker N (%)* (%)** 1722 (34.3) (100) 196 (31.1) (11.4) 1526 (34.8) (88.6)

Smoker N (%)* (%)** 887 (17.7) (100) 92 (14.6) (10.4) 795 (18.1) (89.6)

Overall health status^1^

Poor/not so good N (%)* (%)** 2059 (40.7) (100) 341 (52.9) (16.6) 1718 (38.9) (83.4) < 0.001^c^

Good/very good N (%)* (%)** 2998 (59.3) (100) 304 (47.1) (10.1) 2694 (61.1) (89.9)

Circulatory diseases^1, 2^ N (%)* (%)** 624 (11.8) (100) 118 (17.5) (18.9) 506 (11.0) (81.1) < 0.001^c^

Respiratory diseases^1, 3^ N (%)* (%)** 819 (15.5) (100) 116 (17.2) (14.2) 703 (15.3) (85.8) 0.197^c^

Kidney disease^1^ N (%)* (%)** 189 (3.6) (100) 31 (4.6) (16.4) 158 (3.4) (83.6) 0.129^c^

Diabetes ^1^ N (%)* (%)** 410 (7.8) (100) 75 (11.1) (18.3) 335 (7.3) (81.7) < 0.001^c^

Cancer^1^ N (%)* (%)** 650 (12.3) (100) 85 (12.6) (13.1) 565 (12.3) (86.9) 0.807^c^

Musculoskeletal diseases^1, 4^ N (%)* (%)** 2648 (52.9) (100) 367 (56.6) (13.9) 2281 (52.3) (86.1) 0.041^c^

**Overall** **Non-drinkers**  **Current drinkers^a^ p-value**

**last year^a^**

HADS anxiety Mean (SD) 4.3 (3.3) 4.6 (3.7) 4.2 (3.2)

Median (range) 4 (0-19) 4 (0-19) 4 (0-19) 0.296^b^

HADS depression Mean (SD) 3.8 (2.9) 4.3 (3.2) 3.8 (2.8)

Median (range) 3 (0-18) 4 (0-16) 3 (0-18) 0.001^b^

Drugs with addiction potential^5^

BZD, z-hypnotics or opioids N (%)* (%)** 2174 (41.1) (100) 338 (50.0) (15.5) 1836 (39.8) (84.5) < 0.001^c^

BZD or z-hypnotics N (%)* (%)** 1852 (35.0) (100) 291 (43.0) (15.7) 1561 (33.9) (84.3) < 0.001^c^

BZD N (%)* (%)** 821 (15.5) (100) 153 (22.6) (18.6) 668 (14.5) (81.4) < 0.001^c^

Z-hypnotics N (%)* (%)** 1363 (25.8) (100) 193 (28.6) (14.2) 1170 (25.4) (85.8) 0.080^c^

Opioids N (%)* (%)** 766 (14.5) (100) 133 (19.7) (17.4) 633 (13.7) (82.6) < 0.001^c^

HADS = Hospital Anxiety and Depression Scale; BZD = benzodiazepines

*Column percent

**Row percent

^1^Number do not sum up to 5,284 because of missing information.

^2^Circulatory diseases defined as self-reported myocardial infarction, heart failure, stroke or brain haemorrhage.

^3^Respiratory diseases defined as self-reported asthma, chronic bronchitis, emphysema or chronic obstructive pulmonary disease.

^4^Musculoskeletal diseases defined as self-reported arthritis, rheumatoid arthritis, Bechterew’s disease, osteoporosis, fibromyalgia, degenerative joint disease or osteoarthritis.

^5^Information about prescribed drugs with addiction potential among participants in HUNT3 (2006-08) was drawn from the Norwegian Prescription Database. Drugs with addiction potential were defined as at least one prescription of benzodiazepines, z-hypnotics or opioids in two consecutive years (2005/2006, 2006/2007, 2007/2008 or 2008/2009). Benzodiazepines defined by N03AE, N05BA and N05CD. Z-hypnotics defined by N05CF. Opioids defined by N02A.

^a^Self-reported alcohol consumption assessed among participants in HUNT3. Current drinkers defined as drinking at least a few times a year.

^b^Significance testing with Mann-Whitney U test between non-drinkers last year and current drinkers.

^c^Significance testing with Chi-square test between non-drinkers last year and current drinkers.
