# Supplementary material for: Mortality in older adults with frequent alcohol consumption and use of drugs with addiction potential – The Nord Trøndelag Health Study 2006-2008 (HUNT3), Norway, a population-based study
Source: PLoS One. 2019 Apr 16;14(4):e0214813. doi: 10.1371/journal.pone.0214813 (PMC6467384; doi:10.1371/journal.pone.0214813)
Supplement: S6 Table — Never drinkers excluded. The HUNT Study 2006–08 (HUNT3). (DOCX) [file pone.0214813.s006.docx]

**S6 Table: Overall sample characteristics and according to drinking status (non-drinkers last year versus current drinkers) in older Norwegian men (≥ 65 years, N = 5,227). Never drinkers excluded. The HUNT Study 2006-08 (HUNT3)**

**Overall** **Non-drinkers**  **Current drinkers^a^ p-value**

**last year^a^**

Overall N (%) 5227 (100) 413 (7.9) 4814 (92.1)

Age Mean (SD) 73.4 (6.1) 76.3 (7.0) 73.1 (6.0)

Median (range) 72.4 (65-100.8) 75.2 (65-100.8) 72.1 (65-97.7) < 0.001^b^

Age category

65-74 years N (%)* (%)** 3346 (64.0) (100) 194 (47.0) (5.8) 3152 (65.5) (94.2) < 0.001^c^

≥ 75 years N (%)* (%)** 1881 (36.0) (100) 219 (53.0) (11.6) 1662 (34.5) (88.4)

Level of education^1^

Up to ten years education N (%)* (%)** 3657 (80.1) (100) 298 (86.6) (8.1) 3359 (79.5) (91.9) 0.007^c^

Vocational and general N (%)* (%)** 137 (3.0) (100) 7 (2.0) (5.1) 130 (3.1) (94.9)

College and university N (%)* (%)** 773 (16.9) (100) 39 (11.4) (5.0) 734 (17.4) (95.0)

Residence^1^

Urban N (%)* (%)** 3153 (61.0) (100) 224 (54.5) (7.1) 2929 (61.5) (92.9) 0.005^c^

Rural N (%)* (%)** 2020 (39.0) (100) 187 (45.5) (9.3) 1833 (38.5) (90.7)

Marital status^1^

No living spouse or partner N (%)* (%)** 1246 (23.8) (100) 122 (29.5) (9.8) 1124 (23.4) (90.2) 0.005^c^

Living spouse or partner N (%)* (%)** 3979 (76.2) (100) 291 (70.5) (7.3) 3688 (76.6) (92.7)

Smoking status^1^

Never smoked N (%)* (%)** 1309 (25.7) (100) 110 (27.6) (8.4) 1199 (25.5) (91.6) 0.156^c^

Former smoker N (%)* (%)** 2859 (56.0) (100) 230 (57.6) (8.0) 2629 (55.9) (92.0)

Smoker N (%)* (%)** 933 (18.3) (100) 59 (14.8) (6.3) 874 (18.6) (93.7)

Overall health status^1^

Poor/not so good N (%)* (%)** 1806 (35.5) (100) 200 (50.1) (11.1) 1606 (34.3) (88.9) < 0.001^c^

Good/very good N (%)* (%)** 3279 (64.5) (100) 199 (49.9) (6.1) 3080 (65.7) (93.9)

Circulatory diseases^1, 2^ N (%)* (%)** 1181 (22.6) (100) 128 (31.0) (10.8) 1053 (21.9) (89.2) < 0.001^c^

Respiratory diseases^1, 3^ N (%)* (%)** 800 (15.3) (100) 70 (16.9) (8.8) 730 (15.2) (91.2) 0.339^c^

Kidney disease^1^ N (%)* (%)** 237 (4.5) (100) 23 (5.6) (9.7) 214 (4.4) (90.3) 0.287^c^

Diabetes ^1^ N (%)* (%)** 523 (10.0) (100) 56 (13.6) (10.7) 467 (9.7) (89.3) 0.012^c^

Cancer^1^ N (%)* (%)** 674 (12.9) (100) 64 (15.5) (9.5) 610 (12.7) (90.5) 0.097^c^

Musculoskeletal diseases^1, 4^ N (%)* (%)** 1310 (26.6) (100) 131 (34.0) (10.0) 1179 (26.0) (90.0) 0.001^c^

**Overall** **Non-drinkers**  **Current drinkers^a^ p-value**

**last year^a^**

HADS anxiety Mean (SD) 3.2 (2.7) 3.7 (3.1) 3.1 (2.7)

Median (range) 3 (0-18) 3 (0-18) 3 (0-17) 0.010^b^

HADS depression Mean (SD) 4.2 (2.9) 5.2 (3.3) 4.1 (2.9)

Median (range) 4 (0-17) 5 (0-15) 4 (0-17) < 0.001^b^

Drugs with addiction potential^5^

BZD, z-hypnotics or opioids N (%)* (%)** 1206 (23.1) (100) 121 (29.3) (10.0) 1085 (22.5) (90.0) 0.002^c^

BZD or z-hypnotics N (%)* (%)** 873 (16.7) (100) 93 (22.5) (10.7) 780 (16.2) (89.3) 0.001^c^

BZD N (%)* (%)** 376 (7.2) (100) 53 (12.8) (14.1) 323 (6.7) (85.9) < 0.001^c^

Z-hypnotics N (%)* (%)** 630 (12.1) (100) 58 (14.0) (9.2) 572 (11.9) (90.8) 0.195^c^

Opioids N (%)* (%)** 530 (10.1) (100) 57 (13.8) (10.8) 473 (9.8) (89.2) 0.010^c^

HADS = Hospital Anxiety and Depression Scale; BZD = benzodiazepines

*Column percent

**Row percent

^1^Number do not sum up to 5,227 because of missing information.

^2^Circulatory diseases defined as self-reported myocardial infarction, heart failure, stroke or brain haemorrhage.

^3^Respiratory diseases defined as self-reported asthma, chronic bronchitis, emphysema or chronic obstructive pulmonary disease.

^4^Musculoskeletal diseases defined as self-reported arthritis, rheumatoid arthritis, Bechterew’s disease, osteoporosis, fibromyalgia, degenerative joint disease or osteoarthritis.

^5^Information about prescribed drugs with addiction potential among participants in HUNT3 (2006-08) was drawn from the Norwegian Prescription Database. Drugs with addiction potential were defined as at least one prescription of benzodiazepines, z-hypnotics or opioids in two consecutive years (2005/2006, 2006/2007, 2007/2008 or 2008/2009). Benzodiazepines defined by N03AE, N05BA and N05CD. Z-hypnotics defined by N05CF. Opioids defined by N02A.

^a^Self-reported alcohol consumption assessed among participants in HUNT3. Non-drinkers last year defined as those reporting not drinking alcohol at all last year. Current drinkers defined as drinking at least a few times a year.

^b^Significance testing with Mann-Whitney U test between non-drinkers last year and current drinkers.

^c^Significance testing with Chi-square test between non-drinkers last year and current drinkers.
