# Supplementary material for: Mortality in older adults with frequent alcohol consumption and use of drugs with addiction potential – The Nord Trøndelag Health Study 2006-2008 (HUNT3), Norway, a population-based study
Source: PLoS One. 2019 Apr 16;14(4):e0214813. doi: 10.1371/journal.pone.0214813 (PMC6467384; doi:10.1371/journal.pone.0214813)
Supplement: S7 Table — Never drinkers, non-drinkers last year, those with medical diagnoses at baselinea and those who died within the first year after participation in HUNT3 all excluded. Overall sample characteristics and according to mortality (N = 1,337). The HUNT Study 2006–08 (HUNT3). (DOCX) [file pone.0214813.s007.docx]

**S7 Table: Sensitivity analyses in older Norwegian women (≥ 65 years). Never drinkers, non-drinkers last year, those with medical diagnoses at baseline^a^ and those who died within the first year after participation in HUNT3 all excluded. Overall sample characteristics and according to mortality (N = 1,337). The HUNT Study 2006-08 (HUNT3)**

**Overall** **Alive** **Dead** **p-value**

Overall N (%) 1337 (100) 1245 (93.1) 92 (6.9)

Age Mean (SD) 72.5 (5.9) 72.1 (5.6) 78.3 (7.7)

Median (range) 71.1 (65-95.6) 70.8 (65.1-95.6) 78.9 (65.1-95.6) < 0.001^b^

Age category

65-74 years N (%)* (%)** 919 (68.7) (100) 886 (71.2) (96.4) 33 (35.9) (3.6) < 0.001^c^

≥ 75 years N (%)* (%)** 418 (31.3) (100) 359 (28.8) (85.9) 59 (64.1) (14.1)

Level of education^1^

Up to ten years education N (%)* (%)** 1002 (84.1) (100) 932 (84.0) (93.0) 70 (85.4) (7.0) 0.771^d^

Vocational and general N (%)* (%)** 24 (2.0) (100) 22 (2.0) (91.7) 2 (2.4) (8.3)

College and university N (%)* (%)** 165 (13.9) (100) 155 (14.0) (93.9) 10 (12.2) (6.1)

Residence^1^

Urban N (%)* (%)** 859 (64.9) (100) 803 (65.1) (93.5) 56 (61.5) (6.5) 0.489^c^

Rural N (%)* (%)** 465 (35.1) (100) 430 (34.9) (92.5) 35 (38.5) (7.5)

Marital status^1^

No living spouse or partner N (%)* (%)** 585 (43.8) (100) 530 (42.6) (90.6) 55 (59.8) (9.4) 0.001^c^

Living spouse or partner N (%)* (%)** 751 (56.2) (100) 714 (57.4) (95.1) 37 (40.2) (4.9)

Smoking status^1^

Never smoked N (%)* (%)** 664 (52.1) (100) 628 (52.8) (94.6) 36 (42.4) (5.4) 0.017^c^

Former smoker N (%)* (%)** 379 (29.7) (100) 355 (29.9) (93.7) 24 (28.2) (6.3)

Smoker N (%)* (%)** 231 (18.2) (100) 206 (17.3) (89.2) 25 (29.4) (10.8)

Overall health status^1^

Poor/not so good N (%)* (%)** 216 (16.8) (100) 187 (15.7) (86.6) 29 (32.2) (13.4) < 0.001^c^

Good/very good N (%)* (%)** 1067 (83.2) (100) 1006 (84.3) (94.3) 61 (67.8) (5.7)

HADS anxiety Mean (SD) 3.7 (2.9) 3.6 (2.9) 4.1 (3.4)

Median (range) 3 (0-15) 3 (0-15) 3 (0-15) 0.514^b^

HADS depression Mean (SD) 3.3 (2.6) 3.3 (2.6) 3.9 (2.9)

Median (range) 3 (0-15) 3 (0-15) 3 (0-12) 0.073^b^

**Overall** **Alive** **Dead** **p-value**

Drinking frequency^2^

Few times a year N (%)* (%)** 556 (41.6) (100) 518 (41.6) (93.2) 38 (41.3) (6.8) 0.513^d^

About once a month N (%)* (%)** 170 (12.7) (100) 152 (12.2) (89.4) 18 (19.6) (10.6)

2-3 days a month N (%)* (%)** 225 (16.9) (100) 211 (16.9) (93.8) 14 (15.2) (6.2)

1 day a week N (%)* (%)** 230 (17.2) (100) 216 (17.4) (93.9) 14 (15.2) (6.1)

2-3 days a week N (%)* (%)** 125 (9.3) (100) 118 (9.5) (94.4) 7 (7.6) (5.6)

4-7 days a week N (%)* (%)** 31 (2.3) (100) 30 (2.4) (96.7) 1 (1.1) (3.3)

Drugs with addiction potential^3^

BZD, z-hypnotics or opioids N (%)* (%)** 383 (28.6) (100) 348 (28.0) (90.9) 35 (38.0) (10.1) 0.039^c^

BZD or z-hypnotics N (%)* (%)** 334 (25.0) (100) 308 (24.7) (92.2) 26 (28.3) (7.8) 0.451^c^

BZD N (%)* (%)** 131 (9.8) (100) 122 (9.8) (93.2) 9 (9.8) (6.8) 0.996^c^

Z-hypnotics N (%)* (%)** 247 (18.5) (100) 226 (18.2) (91.5) 21 (22.8) (8.5) 0.265^c^

Opioids N (%)* (%)** 84 (6.3) (100) 67 (5.4) (79.8) 17 (18.5) (20.2) < 0.001^c^

Possible combination of alcohol

consumption ≥ 4 days/week^2^ and

use of prescribed drugs with addiction

potential^3^ N (%)* (%)** 9 (0.7) (100) 9 (0.7) (100) 0 (0.0) (0.0) 1.000^d^

HADS = Hospital Anxiety and Depression Scale; BZD = benzodiazepines

*Column percent

**Row percent

^1^Number do not sum up to 1,337 because of missing information.

^2^Self-reported alcohol consumption assessed among participants in HUNT3.

^3^Information about prescribed drugs with addiction potential among participants in HUNT3 (2006-08) was drawn from the Norwegian Prescription Database. Drugs with addiction potential were defined as at least one prescription of benzodiazepines, z-hypnotics or opioids in two consecutive years (2005/2006, 2006/2007, 2007/2008 or 2008/2009). Benzodiazepines defined by N03AE, N05BA and N05CD. Z-hypnotics defined by N05CF. Opioids defined by N02A.

^a^Excluded participants in HUNT3 with circulatory diseases, respiratory diseases, kidney disease, diabetes, cancer and musculoskeletal diseases at baseline.

^b^Significance testing with Mann-Whitney U test between alive and dead participants (follow-up period from 2006 to 2013).

^c^Significance testing with Chi-square test between alive and dead participants (follow-up period from 2006 to 2013).

^d^Significance testing with Fisher’s Exact test between alive and dead participants (follow-up period from 2006 to 2013).
