# Supplementary material for: Mortality in older adults with frequent alcohol consumption and use of drugs with addiction potential – The Nord Trøndelag Health Study 2006-2008 (HUNT3), Norway, a population-based study
Source: PLoS One. 2019 Apr 16;14(4):e0214813. doi: 10.1371/journal.pone.0214813 (PMC6467384; doi:10.1371/journal.pone.0214813)
Supplement: S10 Table — Never drinkers, non-drinkers last year, those with medical diagnoses at baselinea and those who died within the first year after participation in HUNT3 all excluded. Association between drinking frequency, use of prescribed drugs with addiction potential and all-cause mortality in unadjusted and adjusted logistic regression analyses. The HUNT Study 2006–08 (HUNT3). (DOCX) [file pone.0214813.s010.docx]

**S10 Table: Sensitivity analyses in older Norwegian men (≥ 65 years). Never drinkers, non-drinkers last year, those with medical diagnoses at baseline^a^ and those who died within the first year after participation in HUNT3 all excluded. Association between drinking frequency, use of prescribed drugs with addiction potential and all-cause mortality in unadjusted and adjusted logistic regression analyses. The HUNT Study 2006-08 (HUNT3)**

**Unadjusted^b^ models Adjusted^c^ models Unadjusted^b^ models Adjusted^c^ models Unadjusted^b^ models Adjusted^c^ models**

N = 1783 N = 1224 N = 1309 N = 924 N = 474 N = 300

Overall ≥ 65 years Overall ≥ 65 years 65-74 years 65-74 years ≥ 75 years ≥ 75 years

OR 95% CI OR 95% CI OR 95% CI OR 95% CI OR 95% CI OR 95% CI

**Model 1:**

Drinking frequency^1^

Few times a year 1 (ref) 1 (ref) 1 (ref) 1 (ref) 1 (ref) 1 (ref)

Once a month 0.65 (0.39-1.10) 0.78 (0.38-1.61) 0.71 (0.30-1.67) 0.82 (0.29-2.33) 0.76 (0.38-1.50) 0.63 (0.22-1.76)

2-3 days a month 0.77 (0.50-1.18) 1.51 (0.85-2.67) 1.08 (0.56-2.08) 1.30 (0.57-2.93) 1.01 (0.53-1.91) 1.84 (0.78-4.34)

1 day a week **0.59 (0.38-0.91)** 0.65 (0.34-1.24) 0.67 (0.33-1.33) 0.67 (0.27-1.65) 0.96 (0.53-1.76) 0.58 (0.22-1.50)

2-3 days a week **0.46 (0.27-0.77)** 0.49 (0.22-1.11) 0.63 (0.29-1.37) 0.39 (0.12-1.28) 0.62 (0.29-1.33) 0.63 (0.21-1.93)

4-7 days a week 0.71 (0.34-1.49) 1.22 (0.47-3.17) 0.81 (0.26-2.48) 0.59 (0.12-2.86) 1.26 (0.42-3.79) 2.80 (0.70-11.2)

**Model 2a-2e:**

Drugs with addiction

potential^2^

**Model 2a:**

BZD, z-hypnotics

or opioids **2.00 (1.40-2.88)** **1.76 (1.05-2.93)** **2.50 (1.47-4.28)** **2.96 (1.45-6.08)**  1.23 (0.74-2.05) 1.12 (0.54-2.33)

**Model 2b:**

BZD or z-hypnotics **2.11 (1.41-3.16)** 1.52 (0.84-2.77) **2.06 (1.10-3.85)** 1.58 (0.61-4.06) 1.69 (0.96-2.95) 1.46 (0.66-3.20)

**Model 2c:**

BZD **2.30 (1.28-4.14)** 2.20 (0.92-5.34) 2.36 (0.97-5.73) 2.12 (0.57-7.80) 1.94 (0.83-4.52) 2.25 (0.65-7.71

**Model 2d:**

Z-hypnotics **1.99 (1.26-3.14)** 1.53 (0.80-2.91) 1.83 (0.88-3.79) 1.61 (0.58-4.45) 1.62 (0.87-3.00) 1.49 (0.63-3.52)

**Model 2e:**

Opioids **1.81 (1.07-3.08)** **2.25 (1.12-4.54)** **3.03 (1.48-6.19)** **4.36 (1.83-10.39)**  0.84 (0.38-1.89) 1.01 (0.31-3.26)

**Model 3:**

Possible combination

of alcohol consumption

≥ 4 days a week^1^ and

use of prescribed drugs

with addiction potential^2^ 1.51 (0.44-5.19) 3.65 (0.86-15.54) 1.32 (0.17-10.28) 2.33 (0.28-19.50) 1.33 (0.25-6.95) 8.10 (0.49-134.1)

*Note:* Bold numbers indicate significant associations. OR = odds ratio; CI = confidence interval; BZD = benzodiazepines

^1^Self-reported alcohol consumption assessed among participants in HUNT3.

^2^Information on prescribed drugs with addiction potential among participants in HUNT3 (2006-08) was drawn from the Norwegian prescription database. Drugs with addiction potential were defined as at least one prescription of BZD, z-hypnotics or opioids in one year for a minimum of two consecutive years (2005/2006, 2007/2008 or 2008/2009). BZD defined by N03AE, N05BA and N05CD. Z-hypnotics defined by N05CF. Opioids defined by N02A.

^a^Excluded those with circulatory diseases, respiratory diseases, kidney disease, diabetes, cancer and musculoskeletal diseases at baseline.

^b^Unadjusted binary logistic regression analysis. Dependent variable: All-cause mortality (2006-2013). Exposure variables: Model 1: Drinking frequency (drinking occasionally a few times a year reference category), Model 2a: BZD, z-hypnotics or opioids (no prescribed BZD, z-hypnotics or opioids reference category), Model 2b: BZD or z-hypnotics (no prescribed BZD or z-hypnotics reference category), Model 2c: BZD (no prescribed BZD reference category), Model 2d: z-hypnotics (no prescribed z-hypnotics reference category), Model 2e: opioids (no prescribed opioids reference category), Model 3: possible combination of alcohol consumption ≥ 4 days/week and being prescribed drugs with addiction potential (BZD, z-hypnotics or opioids). Reference category: no alcohol consumption ≥ 4 days/week, no prescribed drugs with addiction potential, or neither alcohol consumption ≥ 4 days/week nor being prescribed drugs with addiction potential.

^c^Adjusted binary logistic regression analysis: Dependent variable: All-cause mortality (2006-2013). Exposure variables: The same as in the unadjusted analyses. Adjusted for gender (women reference category), age (continuous variable), level of education (up to ten year education reference category), living in urban versus rural areas, marital status (no living spouse or partner reference category), smoking status (never smoked reference category), overall health status (poor/not so good reference category), Hospital Anxiety and Depression Scale (HADS) anxiety scale (continuous variable) and HADS depression scale (continuous variable).
